# A genome-wide search for pleiotropy in more than 100,000 harmonized longitudinal cognitive domain scores

**DOI:** 10.1186/s13024-023-00633-4

**Published:** 2023-06-22

**Authors:** Moonil Kang, Ting Fang Alvin Ang, Sherral A. Devine, Richard Sherva, Shubhabrata Mukherjee, Emily H. Trittschuh, Laura E. Gibbons, Phoebe Scollard, Michael Lee, Seo-Eun Choi, Brandon Klinedinst, Connie Nakano, Logan C. Dumitrescu, Alaina Durant, Timothy J. Hohman, Michael L. Cuccaro, Andrew J. Saykin, Walter A. Kukull, David A. Bennett, Li-San Wang, Richard P. Mayeux, Jonathan L. Haines, Margaret A. Pericak-Vance, Gerard D. Schellenberg, Paul K. Crane, Rhoda Au, Kathryn L. Lunetta, Jesse B. Mez, Lindsay A. Farrer

**Affiliations:** 1 Department of Medicine (Biomedical Genetics), Boston University Chobanian & Avedisian School of Medicine, 72 East Concord Street E200, Boston, MA 02118 USA; 2 Department of Anatomy and Neurobiology, Boston University Chobanian & Avedisian School of Medicine, Boston, MA USA; 3 Framingham Heart Study, Boston University Chobanian & Avedisian School of Medicine, Boston, MA USA; 4 Slone Epidemiology Center, Boston University Chobanian & Avedisian School of Medicine, Boston, MA USA; 5 Department of Medicine, University of Washington School of Medicine, Seattle, WA USA; 6 Geriatric Research, Education, and Clinical Center, Veterans Affairs Puget Sound Health Care System, Seattle, WA USA; 7 Department of Psychiatry and Behavioral Sciences, University of Washington School of Medicine, Seattle, WA USA; 8 Vanderbilt Memory & Alzheimer’s Center, Vanderbilt University Medical Center, Nashville, TN USA; 9 Vanderbilt Genetics Institute, Vanderbilt University Medical Center, Nashville, TN USA; 10 John P. Hussman Institute for Human Genomics, Miller School of Medicine, Miami, FL USA; 11 Indiana Alzheimer’s Disease Research Center, Indiana University School of Medicine, Indianapolis, IN USA; 12 Department of Radiology and Imaging Services, Indiana University School of Medicine, Indianapolis, IN USA; 13 Department of Medical and Molecular Genetics, Indiana University School of Medicine, Indianapolis, IN USA; 14 Department of Epidemiology, University of Washington, Seattle, WA USA; 15 Rush Alzheimer’s Disease Center, Rush University Medical Center, Chicago, IL USA; 16 Department of Pathology and Laboratory Medicine, University of Pennsylvania Perelman School of Medicine, Philadelphia, PA USA; 17 Department of Neurology, Columbia University School of Medicine, New York, NY USA; 18 Cleveland Institute for Computational Biology, Department of Population and Quantitative Health Sciences, Case Western Reserve University, Cleveland, OH USA; 19 Boston University Alzheimer’s Disease Research Center, Boston University Chobanian & Avedisian School of Medicine, Boston, MA USA; 20 Department of Epidemiology, Boston University School of Public Health, Boston, MA USA; 21 Department of Biostatistics, Boston University School of Public Health, Boston, MA USA; 22 Department of Neurology, Boston University Chobanian & Avedisian School of Medicine, Boston, MA USA; 23 Department of Ophthalmology, Boston University Chobanian & Avedisian School of Medicine, Boston, MA USA

**Keywords:** Alzheimer’s disease, Genome-wide association study, Cognitive domains, Longitudinal measures, Pleiotropy, Pathway analysis

## Abstract

**Background:**

More than 75 common variant loci account for only a portion of the heritability for Alzheimer’s disease (AD). A more complete understanding of the genetic basis of AD can be deduced by exploring associations with AD-related endophenotypes.

**Methods:**

We conducted genome-wide scans for cognitive domain performance using harmonized and co-calibrated scores derived by confirmatory factor analyses for executive function, language, and memory. We analyzed 103,796 longitudinal observations from 23,066 members of community-based (FHS, ACT, and ROSMAP) and clinic-based (ADRCs and ADNI) cohorts using generalized linear mixed models including terms for SNP, age, SNP × age interaction, sex, education, and five ancestry principal components. Significance was determined based on a joint test of the SNP’s main effect and interaction with age. Results across datasets were combined using inverse-variance meta-analysis. Genome-wide tests of pleiotropy for each domain pair as the outcome were performed using PLACO software.

**Results:**

Individual domain and pleiotropy analyses revealed genome-wide significant (GWS) associations with five established loci for AD and AD-related disorders (*BIN1*, *CR1*, *GRN*, *MS4A6A*, and *APOE*) and eight novel loci. *ULK2* was associated with executive function in the community-based cohorts (rs157405, *P* = 2.19 × 10^–9^). GWS associations for language were identified with *CDK14* in the clinic-based cohorts (rs705353, *P* = 1.73 × 10^–8^) and *LINC02712* in the total sample (rs145012974, *P* = 3.66 × 10^–8^). *GRN* (rs5848, *P* = 4.21 × 10^–8^) and *PURG* (rs117523305, *P* = 1.73 × 10^–8^) were associated with memory in the total and community-based cohorts, respectively. GWS pleiotropy was observed for language and memory with *LOC107984373* (rs73005629, *P* = 3.12 × 10^–8^) in the clinic-based cohorts, and with *NCALD* (rs56162098, *P* = 1.23 × 10^–9^) and *PTPRD* (rs145989094, *P* = 8.34 × 10^–9^) in the community-based cohorts. GWS pleiotropy was also found for executive function and memory with *OSGIN1* (rs12447050, *P* = 4.09 × 10^–8^) and *PTPRD* (rs145989094, *P* = 3.85 × 10^–8^) in the community-based cohorts. Functional studies have previously linked AD to *ULK2*, *NCALD*, and *PTPRD*.

**Conclusion:**

Our results provide some insight into biological pathways underlying processes leading to domain-specific cognitive impairment and AD, as well as a conduit toward a syndrome-specific precision medicine approach to AD. Increasing the number of participants with harmonized cognitive domain scores will enhance the discovery of additional genetic factors of cognitive decline leading to AD and related dementias.

**Supplementary Information:**

The online version contains supplementary material available at 10.1186/s13024-023-00633-4.

## Background

Late-onset Alzheimer’s disease (AD) occurring after age 65 is the most common type of dementia and is highly heritable, estimated at 60% to 80% [1]. Common single-nucleotide polymorphisms (SNPs) explain 24% to 33% of the total phenotypic variance of AD [2–4], of which up to 6% is accounted for by *APOE* [3]. More than 75 loci affecting AD risk have been identified in several large-scale genome-wide association studies (GWAS) [5–10], but much of the underlying genetic architecture of AD remains unknown [11].

Although GWAS conducted in larger samples will undoubtedly reveal additional AD loci, understanding of the genetic influence on AD risk can be improved by examining the association with endophenotypes that potentially highlight specific pathways underlying the complex disease phenotype. Previously, numerous genetic associations have been identified for AD-related endophenotypes, such as cognitive performance [12–15], brain imaging traits [16–18], neuropathological traits [19–21], and biomarkers measured in cerebrospinal fluid [22–24]. GWAS have found several loci for general cognitive ability [13, 25], but most findings for specific cognitive domains are not genome-wide significant (GWS), inconsistent, and rarely replicated in independent datasets, perhaps because of the variability in neuropsychological (NP) tests administered across cohorts [26–29]. To address this concern, Mukherjee and colleagues applied confirmatory factor analysis models to co-calibrate and harmonize composite scores for several cognitive domains. The scores obtained are on the same scale, making them comparable to each other regardless of the NP protocol [15, 30].

Here, we conducted a GWAS for cognitive scores of three domains derived from longitudinal, prospectively collected NP tests administered to participants of several large cohort studies. The statistical power for detecting associations with endophenotypes can be increased by studying outcomes of multiple correlated traits under a model of pleiotropy—a phenomenon where a single gene or variant affects multiple phenotypes [31–34]. This approach has successfully identified novel associations for neuropathological processes in AD [35–37]. Because measures of cognitive performance are highly heritable and correlated with each other [38–40], they are well suited as outcomes for cross-phenotype genetic association studies. Therefore, we also tested pleiotropy models for each pair of the three cognitive domains to identify novel loci which may be involved in AD.

## Methods

### Participants

This study included non-Hispanic white participants of the Framingham Heart Study (FHS), National Institute on Aging sponsored Alzheimer’s Disease Research Centers whose phenotypic information was assembled and curated by the National Alzheimer’s Coordinating Center (NACC), the Adult Changes in Thought (ACT) Study, the Alzheimer’s Disease Neuroimaging Initiative (ADNI), and the Religious Orders Study/Rush Memory and Aging Project (ROSMAP). Briefly, FHS is a long-running multi-generation community-based study of cardiovascular disease and other age-related disorders [41–43], including cognitive decline and dementia [44, 45]. ACT and ROSMAP are also community-based cohorts that recruited unrelated cognitively normal participants who are followed longitudinally for cognitive disorders [46, 47]. Participants of NACC and ADNI were clinically ascertained for AD research and were cognitively normal or met the criteria for mild cognitive impairment (MCI) or AD at the time of enrollment [48–52]. Extensive cognitive testing of participants of all is conducted at all visits. Details regarding the ascertainment, evaluation, and diagnosis of members of these five cohorts were reported elsewhere [45–48, 52].

### Cognitive domain scores

Scores for executive function, language, and memory domains were derived as previously described [15, 30, 53]. Scores are co-calibrated to put them on the same scale regardless of the cognitive battery administered. Briefly, an expert panel of neuropsychologists (EHT, AJS) and a behavioral neurologist (JBM) assigned each NP test item to one of the three domains. Confirmatory factor analysis in Mplus [54] was used for co-calibration. Cognitive data from the most recent visit were first used to derive scores for each domain, with each domain modeled separately. Test items administered in multiple cohorts functioned as anchors for co-calibration. Parameters for anchor items were forced to be the same across studies to put scores across studies on the same metric. Within study, multiple models (including single factor and bifactor models) were considered with the choice of model determined based on a combination of model fit and concordance with neuropsychological theory. Next, each study’s item parameters from calibration of data at the last visit were fixed and used to obtain scores for each person at each time point. Co-calibrated cognitive scores with a standard error (SE) > 0.6 or derived solely from the mini-mental state examination (MMSE), which has a ceiling effect, were excluded. Time points less than age 60 were only available for FHS and were excluded due to concern that cognitive performance under age 60 may have a different genetic architecture that was only being captured in a single study.

### Genotype data processing

We obtained genome-wide SNP data that were processed and imputed using the Trans-Omics for Precision Medicine (TOPMed) reference panel and aligned to Genome Research Consortium human build 38 (GRCh38) [10, 55]. Variants with poor imputation quality (*r*^2^ < 0.3), minor allele frequencies (MAF) < 0.01, call rates < 95%, and Hardy-Weinberg Equilibrium (HWE) test *p*-value < 1 × 10^–6^ were excluded, and approximately nine million variants remained for each cohort after quality control (QC). Principal components (PCs) of population structure were generated for individuals within each cohort using the set of post-QC variants that were pruned on the basis of a linkage disequilibrium (LD) threshold of 0.1 using the R package GENESIS [56]. Measures of relatedness, kinship coefficients for family-based samples and empirical identity by descent (IBD) in the other samples, were estimated using established procedures [57–59].

### Genetic and phenotypic correlation estimation

Genetic correlations between each pair of cognitive domain scores (executive function, language, and memory) were estimated in each cohort using GREML [60, 61]. The kinship matrix derived from self-reported FHS pedigrees was incorporated in the estimates using the kinship2 package [62]. We used the empirical genetic relationship matrix (GRM) to account for relatedness among individuals in the other cohorts. To concurrently investigate SNP associations with both performance at the median age and change in cognitive function over time in each domain, we applied a joint test of the marginal genetic effects and gene × age interaction together in a generalized linear mixed model framework with a random slope and intercept as implemented in the mixed‐model association test for gene-environment interactions (MAGEE) R package [63, 64]. Models included terms for SNP, the interaction between SNP and age, and covariates for age, sex, educational level (less than high school, high school, some college, or college graduate), and the first five PCs represented as follows:

$$factor\, score={\alpha }_{A}age+{\alpha }_{B}sex+{\alpha }_{C}education+{\beta }_{G}SNP+{\gamma }_{X}\left(SNP\times age\right)+{\alpha }_{i}\sum\limits_{i=1}^{5}{PC}_{i}+r$$

where *α*_*A*_, *α*_*B*_, and *α*_*C*_ indicate the effects of age, sex, and educational level, respectively; *β*_*G*_ is the main SNP effect; *γ*_*X*_ represents the SNP × age interaction effect; *α*_*i*_ is the effect of the *i*th PC (*i* between 1 and 5); and *r* is a random intercept. We subtracted the median age for all observations for all individuals in the dataset from the individual’s age at each exam in order to center age because the intercept will refer to the mean outcome value when an individual’s baseline age is equal to the mean age at baseline in each dataset. Models also incorporated the GRM as a random effect.

### Cross-trait analyses

We performed cross-traits LD score regression [65] to estimate genetic correlations across general cognitive function, cognitive domain scores, and neuropsychiatric disorders. We used GWAS summary statistics for general cognitive function (*n* = 300,486) [13], cognitive factor scores (*n* = 23,066) from the current study, neuropsychiatric disorders—AD, bipolar disorder, schizophrenia (*n* = 420,531), and depression (*n* = 370,457)—from the Pan-UK Biobank, and LD scores derived from the 1000 Genomes Project (phase 3) European samples. We only included 4,815,014 variants with imputation quality *r*^2^ > 0.6 and MAF > 0.01 in cross-trait analyses.

### Genetic association analyses

Analyses were performed in each dataset separately, and the GWAS results were combined across datasets by meta-analyses. To correct systematic inflation in a joint test of the SNP’s main and interaction effects [66, 67], we applied the joint meta-analysis method [68] which considers the covariance between the main and interaction effects, and the inverse variance weighted approach in METAL [69]. Meta-analyses were performed for each cognitive domain in the total sample, clinic-based cohorts (NACC and ADNI), and community-based cohorts (FHS, ACT, and ROSMAP) separately (Fig. S1). Results for the clinic- and community-based cohorts were considered separately because some associations might be unique to one of the cohort groups due to disparity in age or proportion of participants with AD. The genomic inflation factor (*λ*) was calculated for each GWAS and applied to adjust *p*-values for each test. A GWS threshold was set at *P* = 5 × 10^–8^.

### Genome-wide pleiotropy analyses

We conducted a pleiotropy GWAS for each pair of cognitive domains in the total sample, clinic-based cohorts, and community-based cohorts using the pooled GWAS results from the joint meta-analysis (Fig. S1) and the R package PLACO [70, 71]. Because rejecting the global null hypothesis that neither phenotype is associated does not specifically imply the existence of pleiotropy, PLACO tests the composite null hypothesis that no more than one phenotype is associated with a variant. Thus, rejecting the composite null hypothesis implies that both phenotypes are associated with the variant, *i*.*e*., pleiotropy. This approach uses the product of the *Z*-statistics as the test statistic for the association of a given variant with each individual trait. The null distribution of the test statistic takes the form of a mixture distribution that allows for the variant to be associated with none or only one of the traits. Variants with squared *Z*-scores > 80 for one trait were removed because they could cause spurious pleiotropic signals [72, 73]. Because correlations between the *Z*-statistics for the association between a variant and the two traits can result in inflated type I errors [74], we adjusted for the Pearson correlation for variants with no effect (*P* > 1 × 10^–4^) as suggested by the developers of the method.

### Pathway enrichment analyses

We performed several pathway analyses, each of which was seeded with genes containing variants associated with a single cognitive domain or pleiotropy for paired domains (*P* < 1 × 10^–4^) in the respective GWAS, using the Ingenuity Pathway Analysis software (QIAGEN Inc.) [75]. Enrichment *p*-values for each canonical pathway were adjusted for a false discovery rate (FDR) using the Benjamini-Hochberg method [76], and an FDR-adjusted *P*-value threshold was set at 0.001 to account for the 18 separate pathway analyses (six single and paired domains multiplied by three sample strata).

## Results

### Cognitive domains are phenotypically and genetically correlated

Compared to the community-based cohorts, the clinic-based cohorts had more males, participants who were younger and better educated, and higher proportion of participants who were diagnosed with MCI or AD (Table 1). Even though the mean and median ages at the last visits of individuals in the clinic-based cohorts were slightly lower than those in the community-based cohorts, scores for executive function and memory were significantly lower (*P* < 0.001), and the language score was significantly higher (*P* < 0.001) in the clinic-based cohorts. Phenotypic and genetic correlations for each pair of factor scores in each dataset were moderate to high (phenotypic *r* = 0.56–0.86, genetic *r* = 0.57–0.72) (Table S1). Most phenotypic and genetic correlations were higher for ROSMAP compared to the other cohorts. Cross-trait analyses revealed that factor scores for all three cognitive domains are significantly genetically correlated with general cognitive function (0.51 ≤ r ≤ 0.77) (Table S2). Although none of the traits were significantly correlated with AD or other psychiatric disorders, the language domain score was moderately associated with depression (*r* = 0.60, *P* = 0.11) and the memory domain score was strongly associated with AD (*r* = 0.90, *P* = 0.40). Lack of significance for these results may be due to insufficient power for genetic correlations with dichotomous outcomes.

**Table 1:** Characteristics of study participants

	**Overall**	**Clinic-based cohorts**			**Community-based cohorts**			
		**NACC**	**ADNI**	**Total**	**FHS**	**ACT**	**ROSMAP**	**Total**
Observations, n	103,796	53,237	7,059	60,296	9,115	15,122	19,263	43,500
Unique participants, n	23,066	12,985	1,367	14,352	3,607	3,035	2,072	8,714
Mean age, years (SD)								
Overall	77.5 ± 8.9	75.7 ± 8.9	76.6 ± 7.4	75.8 ± 8.8	75.2 ± 9.6	79.3 ± 7.2	82.8 ± 7.6	80.0 ± 8.4
First visit	73.1 ± 8.7	72.9 ± 9.0	73.9 ± 7.1	73.0 ± 8.8	69.1 ± 8.3	74.5 ± 6.5	78.8 ± 7.5	73.3 ± 8.4
Last visit	78.2 ± 9.7	76.6 ± 9.3	77.6 ± 7.9	76.6 ± 9.2	75.3 ± 9.9	82.7 ± 7.7	87.8 ± 6.9	80.8 ± 9.9
Median age, years (IQR)								
Overall	78.0 (13.0)	76.0 (12.0)	76.7 (10.0)	76.0 (12.0)	75.0 (16.0)	79.0 (10.0)	83.2 (10.6)	80.4 (12.0)
First visit	73.0 (12.3)	73.0 (12.0)	73.8 (9.8)	73.0 (12.0)	66.0 (11.5)	73.0 (10.0)	79.5 (10.6)	72.0 (14.0)
Last visit	78.0 (14.3)	77.0 (13.0)	77.9 (11.4)	77.0 (13.0)	74.0 (16.0)	83.0 (11.0)	88.2 (9.0)	82.0 (15.6)
Sex, n (%)								
Male	10,198 (44.2)	5,856 (45.1)	766 (56.0)	6,622 (46.1)	1,620 (44.9)	1,331 (43.9)	625 (30.2)	3,576 (41.0)
Female	12,868 (55.8)	7,129 (54.9)	601 (44.0)	7,730 (53.9)	1,987 (55.1)	1,704 (56.1)	1,447 (69.8)	5,138 (59.0)
Educational level, n (%)								
Under high school degree	974 (4.2)	302 (2.3)	41 (3.0)	343 (2.4)	305 (8.5)	240 (7.9)	86 (4.2)	631 (7.2)
High school degree	3,988 (17.3)	1,958 (15.1)	160 (11.7)	2,118 (14.8)	927 (25.7)	620 (20.4)	323 (15.6)	1,870 (21.5)
Some college	4,387 (19.0)	2,212 (17.0)	259 (18.9)	2,471 (17.2)	905 (25.1)	688 (22.7)	323 (15.6)	1,916 (22.0)
Over college graduate	13,717 (59.5)	8,513 (65.6)	907 (66.3)	9,420 (65.6)	1,470 (40.8)	1,487 (49.0)	1,340 (64.7)	4,297 (49.3)
Executive function, mean (SD)								
Overall	0.197 ± 0.752	0.163 ± 0.827	0.318 ± 0.746	0.182 ± 0.819	-0.007 ± 0.688	0.189 ± 0.499	0.343 ± 0.698	0.217 ± 0.649
First visit	0.157 ± 0.748	0.072 ± 0.842	0.377 ± 0.668	0.102 ± 0.832	0.167 ± 0.648	0.248 ± 0.454	0.380 ± 0.598	0.246 ± 0.581
Last visit	-0.091 ± 0.894	-0.143 ± 0.977	0.096 ± 0.891	-0.119 ± 0.971	-0.043 ± 0.726	0.044 ± 0.677	-0.181 ± 0.865	-0.046 ± 0.751
Language, mean (SD)								
Overall	0.353 ± 0.808	0.472 ± 0.869	0.392 ± 0.700	0.463 ± 0.851	0.229 ± 0.662	0.166 ± 0.422	0.154 ± 0.805	0.173 ± 0.695
First visit	0.360 ± 0.739	0.392 ± 0.843	0.467 ± 0.610	0.399 ± 0.824	0.360 ± 0.626	0.239 ± 0.366	0.247 ± 0.625	0.291 ± 0.555
Last visit	0.088 ± 0.935	0.135 ± 1.016	0.186 ± 0.863	0.140 ± 1.003	0.222 ± 0.722	0.039 ± 0.537	-0.429 ± 1.007	-0.004 ± 0.797
Memory, mean (SD)								
Overall	0.337 ± 0.890	0.372 ± 1.007	0.146 ± 0.873	0.346 ± 0.995	0.286 ± 0.636	0.512 ± 0.549	0.194 ± 0.836	0.324 ± 0.721
First visit	0.293 ± 0.798	0.216 ± 0.927	0.235 ± 0.717	0.218 ± 0.909	0.406 ± 0.551	0.590 ± 0.456	0.181 ± 0.573	0.417 ± 0.548
Last visit	0.010 ± 1.042	-0.019 ± 1.155	-0.083 ± 1.035	-0.025 ± 1.144	0.241 ± 0.688	0.176 ± 0.731	-0.393 ± 1.055	0.068 ± 0.845
Cognitive status, n (%)								
Cognitively normal	10,919 (47.3)	4,891 (37.7)	437 (32.0)	5,328 (37.1)	2,740 (76.0)	2,267 (74.7)	584 (28.2)	5,591 (64.2)
MCI	2,248 (9.7)	820 (6.3)	444 (32.5)	1,264 (8.8)	287 (8.0)	-	697 (33.6)	984 (11.3)
AD	9,272 (40.2)	7,090 (54.6)	486 (35.6)	7,576 (52.8)	445 (12.3)	485 (16.0)	766 (37.0)	1,696 (19.5)
Dementia (other than AD)	627 (2.7)	184 (1.4)	-	184 (1.3)	135 (3.7)	283 (9.3)	25 (1.2)	443 (5.1)

### GWAS identifies multiple established AD and novel loci associated with individual cognitive domains

**Fig. 1:**
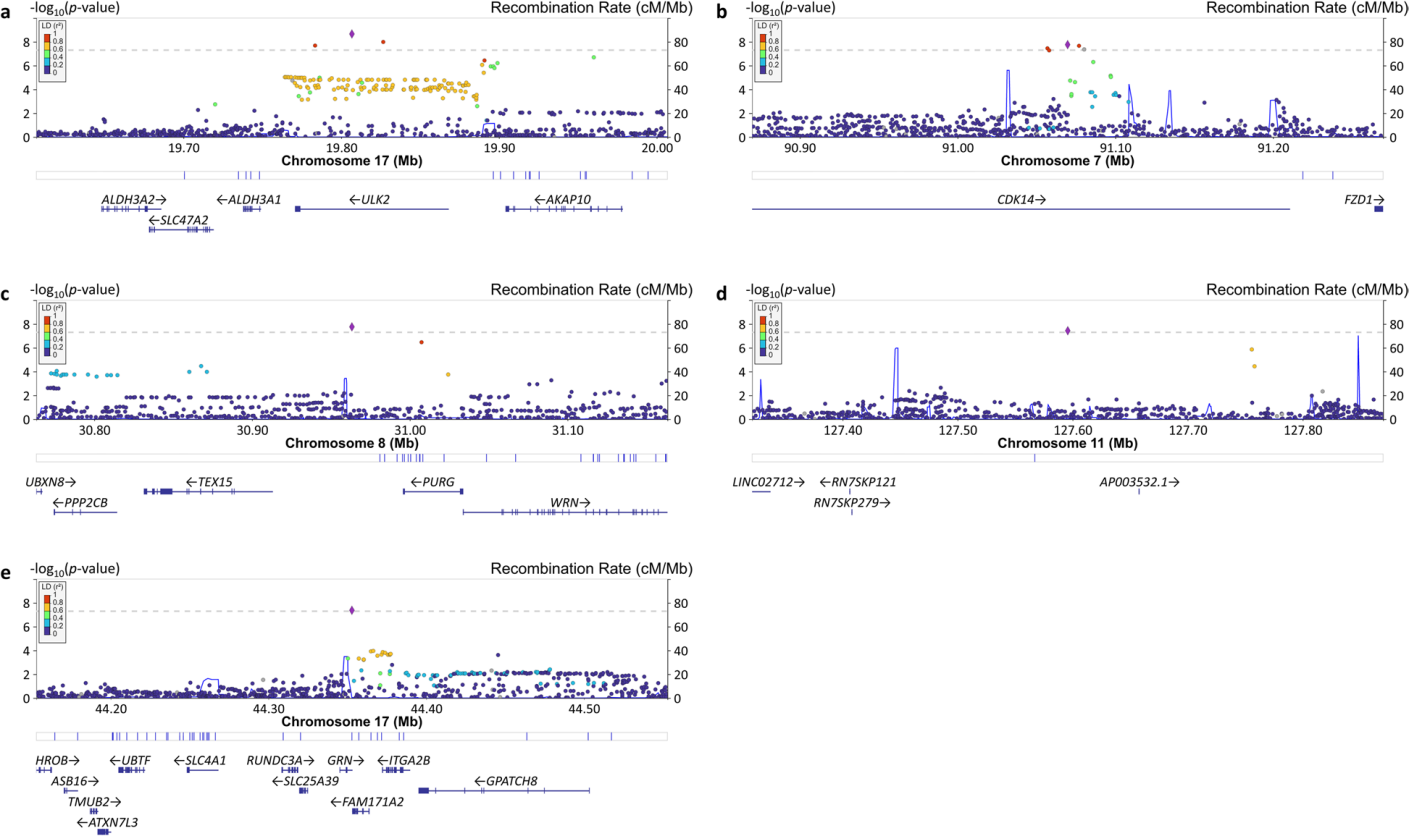
Locus Zoom plots showing the association of SNPs in the regions of novel loci with cognitive domains. The SNP with the lowest *p*-value at each locus is indicated with a purple diamond. Computed estimates of linkage disequilibrium (*r*^2^) of SNPs in the region with top-ranked SNP are color-coded according to the key. Vertical blue lines indicate locations of high recombination rates. Locations of genes in the region are shown below the diagram. **a** Association of rs157405 with executive function in the community-based cohorts. **b** Association of rs705353 with language in the clinic-based cohorts. **c** Association of rs117523305 with memory in the community-based cohorts. **d** Association of rs145012974 with language in the total sample. **e** Association of rs5848 with memory in the total sample

There was little evidence of genomic inflation (*λ* = 1.006–1.023) in the GWAS for each cognitive domain and strata of the sample (Figs. S2, S3 and S4). GWS associations were observed for many SNPs in the *APOE* region for all traits (Table S3). We also identified associations with several other established AD loci (Table 2). *BIN1* SNP rs6733839 was associated with language (*P*_Joint_ = 2.70 × 10^–8^) and memory (*P*_Joint_ = 2.37 × 10^–9^) in the total sample and with both language (*P*_Joint_ = 1.98 × 10^–9^) and memory (*P*_Joint_ = 1.60 × 10^–8^) in the clinic-based cohorts. The significant joint effect of rs6733839 is due primarily to the SNP’s main effect rather than its interaction with age and is supported by multiple adjacent variants (Figs. S5 and S6). GWS associations for memory were also observed with *CR1* SNP rs1752684 (*P*_Joint_ = 8.85 × 10^–9^) and *MS4A6A* SNP rs7232 (*P*_Joint_ = 3.97 × 10^–8^) in the clinic-based cohorts, findings which were supported by adjacent SNPs (Fig. S7). Similar to *BIN1*, results of the joint test of the main and interaction effects for rs1752684 and rs7232 reflect the SNPs’ main effects. GWS associations were also detected with SNPs in four additional loci, *ULK2* (rs157405, *P*_Joint_ = 2.19 × 10^–9^) with executive function in the community-based cohorts, *CDK14* (rs705353, *P*_Joint_ = 1.73 × 10^–8^) with language in the clinic-based cohorts, *PURG* (rs117523305, *P*_Joint_ = 1.73 × 10^–8^) with memory in the community-based cohorts, and *LINC02712* (rs145012974, *P*_Joint_ = 3.66 × 10^–8^) with language in the total sample (Fig. 1). We also identified a GWS association of memory with *GRN* (rs5848, *P*_Joint_ = 4.21 × 10^–8^) in the total sample (Fig. 1). Unlike the associations with the other known AD loci, the interactions of the *ULK2* (*P*_G×Age_ = 7.65 × 10^–7^), *CDK14* (*P*_G×Age_ = 2.54 × 10^–9^), *PURG* (*P*_G×Age_ = 1.41 × 10^–8^), *LINC02712* (*P*_G×Age_ = 7.69 × 10^–9^), and *GRN* (*P*_G×Age_ = 1.07 × 10^–6^) SNPs with age accounted for the significant joint test findings (Table 2).

**Table 2:** Genome-wide significant associations for cognitive domain scores

**Chr**	**Position**	**Variant**	**A1/A2**^**a**^** (MAF)**	**Nearest Gene**	**Cognitive Domain**	**Cohorts**^**b**^	**Genetic effects**				
							***β***_**G**_** (SE)**	***P***_**G**_	***β***_**G×Age**_** (SE)**	***P***_**G×Age**_	***P***_**Joint**_
1	207573951	rs1752684	A/G (0.198)	*CR1*	Executive Function	All	-0.028 (0.007)	1.53 × 10^–4^	-0.002 (0.001)	6.05 × 10^–3^	5.05 × 10^–5^
						Clinic	-0.042 (0.012)	5.91 × 10^–4^	-0.002 (0.001)	7.52 × 10^–2^	6.88 × 10^–4^
						Community	-0.020 (0.009)	2.98 × 10^–2^	-0.002 (0.001)	4.90 × 10^–2^	2.40 × 10^–2^
					Language	All	-0.026 (0.007)	3.90 × 10^–4^	-0.001 (0.001)	6.84 × 10^–2^	6.25 × 10^–4^
						Clinic	-0.054 (0.012)	1.26 × 10^–5^	-0.002 (0.001)	5.07 × 10^–2^	1.63 × 10^–5^
						Community	-0.011 (0.009)	2.14 × 10^–1^	-0.001 (0.001)	5.59 × 10^–1^	4.20 × 10^–1^
					Memory	All	-0.043 (0.008)	1.75 × 10^–7^	-0.002 (0.001)	1.83 × 10^–2^	4.42 × 10^–7^
						Clinic	-0.080 (0.014)	2.44 × 10^–8^	-0.004 (0.001)	1.05 × 10^–2^	**8.85 × 10**^**–9**^
						Community	-0.022 (0.010)	2.46 × 10^–2^	0.000 (0.001)	6.45 × 10^–1^	6.88 × 10^–2^
2	127135234	rs6733839	T/C (0.403)	*BIN1* *CYP27C1*	Executive Function	All	-0.009 (0.006)	1.25 × 10^–1^	0.000 (0.001)	6.54 × 10^–1^	2.92 × 10^–1^
						Clinic	-0.023 (0.010)	1.77 × 10^–2^	0.000 (0.001)	6.84 × 10^–1^	5.58 × 10^–2^
						Community	-0.001 (0.007)	8.70 × 10^–1^	0.000 (0.001)	9.27 × 10^–1^	9.84 × 10^–1^
					Language	All	-0.031 (0.006)	6.90 × 10^–8^	-0.002 (0.001)	3.62 × 10^–3^	**2.70 × 10**^**–8**^
						Clinic	-0.063 (0.010)	4.23 × 10^–10^	-0.001 (0.001)	2.09 × 10^–1^	**1.98 × 10**^**–9**^
						Community	-0.017 (0.007)	1.68 × 10^–2^	-0.002 (0.001)	1.70 × 10^–2^	6.57 × 10^–3^
					Memory	All	-0.039 (0.006)	2.20 × 10^–9^	-0.002 (0.001)	1.62 × 10^–3^	**2.37 × 10**^**–9**^
						Clinic	-0.068 (0.012)	5.23 × 10^–9^	-0.001 (0.001)	2.08 × 10^–1^	**1.60 × 10**^**–8**^
						Community	-0.027 (0.008)	5.71 × 10^–4^	-0.002 (0.001)	1.69 × 10^–2^	1.11 × 10^–3^
7	91069758	rs705353	G/A (0.252)	*CDK14*	Executive Function	All	0.003 (0.006)	6.40 × 10^–1^	-0.002 (0.001)	1.53 × 10^–3^	4.58 × 10^–3^
						Clinic	0.000 (0.011)	9.75 × 10^–1^	-0.005 (0.001)	2.03 × 10^–5^	1.12 × 10^–4^
						Community	0.006 (0.008)	4.20 × 10^–1^	-0.001 (0.001)	4.11 × 10^–1^	4.43 × 10^–1^
					Language	All	0.007 (0.006)	2.68 × 10^–1^	-0.002 (0.001)	5.14 × 10^–4^	5.93 × 10^–4^
						Clinic	-0.004 (0.011)	7.53 × 10^–1^	-0.007 (0.001)	2.54 × 10^–9^	**1.73 × 10**^**–8**^
						Community	0.015 (0.008)	5.28 × 10^–2^	0.000 (0.001)	8.28 × 10^–1^	1.41 × 10^–1^
					Memory	All	-0.003 (0.007)	6.95 × 10^–1^	-0.002 (0.001)	3.03 × 10^–2^	8.72 × 10^–2^
						Clinic	-0.008 (0.013)	5.46 × 10^–1^	-0.005 (0.001)	1.36 × 10^–4^	6.11 × 10^–4^
						Community	0.005 (0.009)	5.93 × 10^–1^	0.000 (0.001)	9.19 × 10^–1^	8.59 × 10^–1^
8	30963282	rs117523305	C/G (0.016)	*PURG* *TEX15*	Executive Function	All	-0.001 (0.022)	9.67 × 10^–1^	-0.006 (0.002)	5.35 × 10^–3^	2.02 × 10^–2^
						Clinic	-0.012 (0.038)	7.42 × 10^–1^	-0.002 (0.004)	5.89 × 10^–1^	8.23 × 10^–1^
						Community	0.003 (0.026)	9.13 × 10^–1^	-0.008 (0.003)	2.65 × 10^–3^	9.18 × 10^–3^
					Language	All	-0.018 (0.021)	4.02 × 10^–1^	-0.008 (0.002)	8.23 × 10^–4^	3.01 × 10^–3^
						Clinic	0.028 (0.039)	4.80 × 10^–1^	-0.001 (0.004)	7.03 × 10^–1^	7.11 × 10^–1^
						Community	-0.041 (0.025)	1.19 × 10^–1^	-0.011 (0.003)	9.16 × 10^–5^	1.87 × 10^–4^
					Memory	All	-0.069 (0.024)	5.27 × 10^–3^	-0.010 (0.002)	5.18 × 10^–5^	3.62 × 10^–5^
						Clinic	-0.041 (0.044)	3.60 × 10^–1^	0.004 (0.004)	3.34 × 10^–1^	3.88 × 10^–1^
						Community	-0.098 (0.029)	7.87 × 10^–4^	-0.018 (0.003)	1.41 × 10^–8^	**1.73 × 10**^**–8**^
11	60173126	rs7232	A/T (0.361)	*MS4A6A*	Executive Function	All	0.012 (0.006)	3.46 × 10^–2^	0.002 (0.001)	2.90 × 10^–3^	2.38 × 10^–3^
						Clinic	0.021 (0.010)	3.46 × 10^–2^	0.000 (0.001)	8.60 × 10^–1^	1.06 × 10^–1^
						Community	0.009 (0.007)	2.07 × 10^–1^	0.002 (0.001)	6.28 × 10^–4^	2.09 × 10^–3^
					Language	All	0.016 (0.006)	6.40 × 10^–3^	0.002 (0.001)	1.04 × 10^–4^	3.61 × 10^–5^
						Clinic	0.045 (0.010)	1.40 × 10^–5^	0.002 (0.001)	4.91 × 10^–2^	1.55 × 10^–5^
						Community	0.003 (0.007)	6.38 × 10^–1^	0.002 (0.001)	1.93 × 10^–3^	6.31 × 10^–3^
					Memory	All	0.027 (0.006)	5.19 × 10^–5^	0.003 (0.001)	2.60 × 10^–5^	9.43 × 10^–7^
						Clinic	0.068 (0.012)	1.25 × 10^–8^	0.001 (0.001)	2.27 × 10^–1^	**3.97 × 10**^**–8**^
						Community	0.011 (0.008)	1.50 × 10^–1^	0.003 (0.001)	4.62 × 10^–4^	1.80 × 10^–3^
11	127595711	rs145012974	A/G (0.014)	*LINC02712* *LINC02098*	Executive Function	All	-0.040 (0.024)	1.03 × 10^–1^	-0.007 (0.002)	4.72 × 10^–3^	7.25 × 10^–3^
						Clinic	-0.013 (0.041)	7.53 × 10^–1^	-0.004 (0.004)	3.14 × 10^–1^	5.72 × 10^–1^
						Community	-0.056 (0.030)	6.46 × 10^–2^	-0.008 (0.003)	5.11 × 10^–3^	6.48 × 10^–3^
					Language	All	-0.029 (0.024)	2.29 × 10^–1^	-0.014 (0.002)	7.69 × 10^–9^	**3.66 × 10**^**–8**^
						Clinic	-0.026 (0.041)	5.31 × 10^–1^	-0.012 (0.004)	2.16 × 10^–3^	7.23 × 10^–3^
						Community	-0.033 (0.029)	2.70 × 10^–1^	-0.016 (0.003)	1.00 × 10^–6^	3.29 × 10^–6^
					Memory	All	-0.052 (0.026)	5.08 × 10^–2^	-0.008 (0.003)	2.46 × 10^–3^	3.99 × 10^–3^
						Clinic	-0.051 (0.047)	2.87 × 10^–1^	-0.010 (0.005)	3.06 × 10^–2^	5.42 × 10^–2^
						Community	-0.050 (0.032)	1.24 × 10^–1^	-0.007 (0.003)	3.00 × 10^–2^	6.31 × 10^–2^
17	19806731	rs157405	G/A (0.023)	*ULK2*	Executive Function	All	0.063 (0.018)	8.61 × 10^–4^	0.008 (0.002)	1.04 × 10^–5^	1.49 × 10^–6^
						Clinic	-0.024 (0.032)	4.62 × 10^–1^	0.002 (0.003)	4.25 × 10^–1^	5.20 × 10^–1^
						Community	0.107 (0.023)	3.21 × 10^–6^	0.011 (0.002)	7.65 × 10^–7^	**2.19 × 10**^**–9**^
					Language	All	0.029 (0.018)	1.19 × 10^–1^	0.002 (0.002)	2.04 × 10^–1^	1.70 × 10^–1^
						Clinic	-0.040 (0.033)	2.29 × 10^–1^	0.002 (0.003)	4.81 × 10^–1^	3.35 × 10^–1^
						Community	0.059 (0.022)	8.39 × 10^–3^	0.003 (0.002)	2.54 × 10^–1^	2.21 × 10^–2^
					Memory	All	0.046 (0.020)	2.78 × 10^–2^	0.001 (0.002)	5.87 × 10^–1^	8.07 × 10^–2^
						Clinic	-0.005 (0.038)	9.07 × 10^–1^	0.002 (0.004)	5.39 × 10^–1^	8.14 × 10^–1^
						Community	0.064 (0.024)	9.43 × 10^–3^	0.001 (0.003)	6.48 × 10^–1^	2.71 × 10^–2^
17	44352876	rs5848	T/C (0.305)	*GRN*	Executive Function	All	-0.007 (0.006)	2.53 × 10^–1^	-0.002 (0.001)	1.32 × 10^–4^	5.43 × 10^–4^
						Clinic	0.003 (0.010)	7.50 × 10^–1^	-0.003 (0.001)	4.56 × 10^–3^	1.67 × 10^–2^
						Community	-0.012 (0.008)	1.14 × 10^–1^	-0.002 (0.001)	5.76 × 10^–3^	1.15 × 10^–2^
					Language	All	-0.012 (0.006)	5.99 × 10^–2^	-0.002 (0.001)	2.86 × 10^–3^	3.53 × 10^–3^
						Clinic	-0.015 (0.011)	1.71 × 10^–1^	-0.001 (0.001)	4.67 × 10^–1^	3.11 × 10^–1^
						Community	-0.011 (0.007)	1.34 × 10^–1^	-0.003 (0.001)	1.56 × 10^–3^	3.43 × 10^–3^
					Memory	All	-0.029 (0.007)	2.19 × 10^–5^	-0.003 (0.001)	1.07 × 10^–6^	**4.21 × 10**^**–8**^
						Clinic	-0.032 (0.012)	9.58 × 10^–3^	-0.003 (0.001)	3.30 × 10^–3^	5.28 × 10^–4^
						Community	-0.028 (0.008)	8.11 × 10^–4^	-0.003 (0.001)	1.29 × 10^–4^	6.50 × 10^–5^
19^c^	44908684	rs429358	C/T (0.231)	*APOE*	Executive Function	All	-0.192 (0.008)	6.75 × 10^–135^	-0.009 (0.001)	1.26 × 10^–28^	**8.58 × 10**^**–145**^
						Clinic	-0.308 (0.011)	5.51 × 10^–166^	-0.010 (0.001)	1.71 × 10^–19^	**1.86 × 10**^**–170**^
						Community	-0.089 (0.010)	5.21 × 10^–17^	-0.006 (0.001)	4.58 × 10^–9^	**7.05 × 10**^**–20**^
					Language	All	-0.193 (0.008)	1.81 × 10^–137^	-0.011 (0.001)	5.99 × 10^–45^	**2.99 × 10**^**–158**^
						Clinic	-0.334 (0.011)	7.15 × 10^–187^	-0.016 (0.001)	2.15 × 10^–46^	**3.89 × 10**^**–206**^
						Community	-0.079 (0.010)	2.00 × 10^–14^	-0.006 (0.001)	5.59 × 10^–7^	**2.52 × 10**^**–16**^
					Memory	All	-0.323 (0.008)	2.16 × 10^–298^	-0.017 (0.001)	2.19 × 10^–76^	**7.51 × 10**^**–321**^
						Clinic	-0.536 (0.013)	2.38 × 10^–359^	-0.017 (0.001)	8.14 × 10^–38^	**3.31 × 10**^**–374**^
						Community	-0.168 (0.011)	3.45 × 10^–47^	-0.012 (0.001)	1.00 × 10^–22^	**4.04 × 10**^**–51**^
19^c^	44908822	rs7412	T/C (0.059)	*APOE*	Executive Function	All	0.063 (0.011)	2.78 × 10^–8^	0.005 (0.001)	5.40 × 10^–6^	**8.28 × 10**^**–11**^
						Clinic	0.180 (0.020)	2.98 × 10^–18^	0.002 (0.002)	2.73 × 10^–1^	**1.42 × 10**^**–17**^
						Community	0.016 (0.013)	2.39 × 10^–1^	0.005 (0.001)	6.71 × 10^–5^	2.88 × 10^–4^
					Language	All	0.055 (0.011)	9.77 × 10^–7^	0.004 (0.001)	1.40 × 10^–4^	**2.93 × 10**^**–8**^
						Clinic	0.194 (0.021)	1.23 × 10^–19^	0.007 (0.002)	1.95 × 10^–4^	**1.19 × 10**^**–21**^
						Community	0.001 (0.013)	9.51 × 10^–1^	0.002 (0.001)	2.35 × 10^–1^	4.67 × 10^–1^
					Memory	All	0.105 (0.012)	9.64 × 10^–17^	0.008 (0.001)	1.11 × 10^–9^	**1.11 × 10**^**–19**^
						Clinic	0.302 (0.025)	5.81 × 10^–34^	0.005 (0.002)	2.09 × 10^–2^	**1.18 × 10**^**–34**^
						Community	0.040 (0.015)	6.93 × 10^–3^	0.006 (0.002)	9.90 × 10^–5^	1.73 × 10^–4^

^a^*A1* Minor allele, *A2* Reference allele, *MAF* Minor allele frequency

^b^*All* Total sample, *Clinic* Clinic-based cohorts, *Community* Community-based cohorts

^c^We presented GWAS results for the *APOE* SNPs—*ε*4 (rs429358) and *ε*2 (rs7412) alleles—but excluded all the other results in the *APOE* region

The *APOE* region comprised many variants significantly associated with all the cognitive domains in all cohort groupings (Table S3). The high LD between these variants suggests that there are not multiple independent association signals, a conclusion supported by evidence that no genes in this region other than the *APOE* account for the observed association with AD risk or onset age [77]. Focusing on the *APOE* SNPs encoding the *ε*4 (rs429358) and *ε*2 (rs7412) alleles, the *ε*4 SNP was significantly associated with lower (worse) scores for all cognitive domains in an age-dependent manner, based on the negative sign of *β*_G×Age_. Notably, the magnitude of the effect of the *ε*4 SNP on memory was approximately 1.7 times greater than on executive function or language in the total sample at the median age (Table 2). In the same sample, the effect of interaction between *ε*4 and age was 1.5–1.9 times larger for memory compared to executive function or language. Conversely, the *ε*2 SNP was significantly associated with higher (better) cognitive domain scores in the clinic-based cohorts at the median age with some limited age-dependent effect (Table 2).

Numerous highly suggestive associations (*P* < 1 × 10^–6^), including several that were nearly GWS (*P* < 1 × 10^–7^), were found for individual cognitive domains with other loci (Table S3). Notably, language was associated with 18 *ADCY2* SNPs in the community-based cohorts (top SNP: rs7734697, *P*_Joint_ = 6.34 × 10^–8^) and with 19 *DAPK2* SNPs in the clinic-based cohorts (top SNP: rs112972763, *P*_Joint_ = 7.76 × 10^–8^). Six *PLXDC2* SNPs were associated with executive function in the total sample (top SNP: rs7083449, *P*_Joint_ = 7.02 × 10^–8^), and most of the evidence was derived from the community-based cohorts.

### Genome-wide pleiotropy analysis identifies the association of cognitive domains with the progranulin gene and four novel loci

GWAS for the three pairs of cognitive domains collectively identified GWS evidence of association with SNPs in five independent loci (Table 3, Table S4) with little evidence of genomic inflation in the total sample or separately within the clinic-based and community-based cohorts (*λ* = 0.964–0.994, Figs. S8, S9 and S10). Consistent with the findings from analyses of individual cognitive domains, GWS evidence of pleiotropy was found for the association of the *APOE* region SNPs with all cognitive domain pairs in the total sample and the clinic-based and community-based cohorts (Table S4). GWS pleiotropy was also observed with rs6733839, located between *BIN1* and *CYP27C1*, in the total sample (*P*_Joint_ = 9.01 × 10^–12^) and the clinic-based cohorts (*P*_Joint_ = 6.85 × 10^–10^) for language and memory (Table 3). The association with rs6733839 was evident in the community-based cohorts (*P*_Joint_ = 2.52 × 10^–4^), strengthened in the total sample (*P*_Joint_ = 9.01 × 10^–12^), well supported by association with neighboring variants (Fig. S11), and attributable primarily to its main effect for each domain (Table 3).

**Table 3:** Genome-wide significant pleiotropic loci for each pair of cognitive domains (excluding the *APOE* region)

**Chr**	**Position**	**Variant**	**A1/A2**^**a**^** (MAF)**	**Nearest Gene**	**Cognitive Domain**	**Individual locus**					**Pleiotropy**		
						***β***_**G**_** (SE)**	***P***_**G**_	***β***_**G×Age**_** (SE)**	***P***_**G×Age**_	***P***_**Joint**_	***P***_**Placo, G**_	***P***_**Placo, G×Age**_	***P***_**Placo, Joint**_
**Total sample**													
2	127135234	rs6733839	T/C (0.403)	*BIN1* *CYP27C1*	Language	-0.031 (0.006)	6.90 × 10^–8^	-0.002 (0.001)	3.62 × 10^–3^	2.70 × 10^–8^	2.72 × 10^–10^	9.11 × 10^–4^	**9.01 × 10**^**–12**^
					Memory	-0.039 (0.006)	2.20 × 10^–9^	-0.002 (0.001)	1.62 × 10^–3^	2.37 × 10^–9^			
4	179150192	rs73005629	C/T (0.022)	*LOC107984373*	Language	-0.048 (0.019)	1.21 × 10^–2^	-0.007 (0.002)	2.06 × 10^–4^	1.19 × 10^–4^	3.69 × 10^–3^	9.32 × 10^–4^	6.16 × 10^–5^
					Memory	-0.062 (0.021)	3.97 × 10^–3^	-0.006 (0.002)	8.12 × 10^–3^	2.11 × 10^–3^			
8	102060792	rs56162098	C/T (0.138)	*NCALD*	Language	0.019 (0.008)	1.64 × 10^–2^	0.001 (0.001)	1.84 × 10^–1^	3.08 × 10^–2^	5.85 × 10^–5^	5.74 × 10^–3^	6.86 × 10^–6^
					Memory	-0.013 (0.009)	1.68 × 10^–1^	-0.001 (0.001)	1.71 × 10^–1^	2.17 × 10^–1^			
9	10155013	rs145989094	A/T (0.013)	*PTPRD*	Exec Function	-0.056 (0.026)	3.22 × 10^–2^	-0.006 (0.002)	1.26 × 10^–2^	1.04 × 10^–2^	2.70 × 10^–2^	2.79 × 10^–3^	9.93 × 10^–4^
					Memory	-0.058 (0.028)	4.56 × 10^–2^	-0.008 (0.003)	3.14 × 10^–3^	5.92 × 10^–3^			
9	10155013	rs145989094	A/T (0.013)	*PTPRD*	Language	-0.093 (0.025)	3.40 × 10^–4^	-0.004 (0.003)	9.29 × 10^–2^	8.84 × 10^–4^	1.22 × 10^–2^	3.47 × 10^–2^	4.65 × 10^–3^
					Memory	-0.058 (0.028)	4.56 × 10^–2^	-0.008 (0.003)	3.14 × 10^–3^	5.92 × 10^–3^			
16	83947805	rs12447050	C/T (0.400)	*OSGIN1* *MLYCD*	Exec Function	0.016 (0.006)	4.70 × 10^–3^	0.002 (0.001)	4.01 × 10^–4^	1.03 × 10^–4^	2.03 × 10^–3^	9.76 × 10^–4^	3.72 × 10^–5^
					Memory	0.018 (0.006)	4.92 × 10^–3^	0.002 (0.001)	9.28 × 10^–3^	2.61 × 10^–3^			
**Clinic-based cohorts**													
2	127135234	rs6733839	T/C (0.409)	*BIN1* *CYP27C1*	Language	-0.063 (0.010)	4.23 × 10^–10^	-0.001 (0.001)	2.09 × 10^–1^	1.98 × 10^–9^	1.78 × 10^–10^	2.20 × 10^–1^	**6.85 × 10**^**–10**^
					Memory	-0.068 (0.012)	5.23 × 10^–9^	-0.001 (0.001)	2.08 × 10^–1^	1.60 × 10^–8^			
4	179150192	rs73005629	C/T (0.022)	*LOC107984373*	Language	-0.112 (0.033)	7.70 × 10^–4^	-0.014 (0.003)	1.68 × 10^–5^	4.66 × 10^–7^	2.06 × 10^–4^	5.11 × 10^–6^	**3.12 × 10**^**–8**^
					Memory	-0.149 (0.038)	1.13 × 10^–4^	-0.015 (0.004)	4.04 × 10^–5^	1.47 × 10^–7^			
8	102060792	rs56162098	C/T (0.138)	*NCALD*	Language	0.006 (0.014)	6.89 × 10^–1^	0.000 (0.001)	7.49 × 10^–1^	8.81 × 10^–1^	7.74 × 10^–1^	9.29 × 10^–1^	9.56 × 10^–1^
					Memory	0.009 (0.017)	5.73 × 10^–1^	0.001 (0.002)	3.54 × 10^–1^	5.62 × 10^–1^			
9	10155013	rs145989094	A/T (0.013)	*PTPRD*	Exec Function	0.016 (0.044)	7.25 × 10^–1^	0.002 (0.004)	7.27 × 10^–1^	8.85 × 10^–1^	4.38 × 10^–1^	8.92 × 10^–1^	7.33 × 10^–1^
					Memory	0.081 (0.053)	1.28 × 10^–1^	0.000 (0.005)	9.83 × 10^–1^	3.08 × 10^–1^			
9	10155013	rs145989094	A/T (0.013)	*PTPRD*	Language	0.026 (0.046)	5.78 × 10^–1^	0.002 (0.004)	6.27 × 10^–1^	7.68 × 10^–1^	5.27 × 10^–1^	7.63 × 10^–1^	7.82 × 10^–1^
					Memory	0.081 (0.053)	1.28 × 10^–1^	0.000 (0.005)	9.83 × 10^–1^	3.08 × 10^–1^			
16	83947805	rs12447050	C/T (0.404)	*OSGIN1* *MLYCD*	Exec Function	0.005 (0.010)	6.31 × 10^–1^	0.001 (0.001)	4.59 × 10^–1^	6.83 × 10^–1^	8.02 × 10^–1^	3.63 × 10^–1^	6.41 × 10^–1^
					Memory	0.001 (0.012)	9.56 × 10^–1^	0.000 (0.001)	7.65 × 10^–1^	9.54 × 10^–1^			
**Community-based cohorts**													
2	127135234	rs6733839	T/C (0.388)	*BIN1* *CYP27C1*	Language	-0.017 (0.007)	1.68 × 10^–2^	-0.002 (0.001)	1.70 × 10^–2^	6.57 × 10^–3^	1.99 × 10^–3^	8.10 × 10^–3^	2.52 × 10^–4^
					Memory	-0.027 (0.008)	5.71 × 10^–4^	-0.002 (0.001)	1.69 × 10^–2^	1.11 × 10^–3^			
4	179150192	rs73005629	C/T (0.021)	*LOC107984373*	Language	-0.011 (0.023)	6.32 × 10^–1^	-0.003 (0.002)	2.25 × 10^–1^	4.48 × 10^–1^	8.73 × 10^–1^	2.86 × 10^–1^	5.58 × 10^–1^
					Memory	-0.006 (0.026)	8.24 × 10^–1^	0.000 (0.003)	8.79 × 10^–1^	9.42 × 10^–1^			
8	102060792	rs56162098	C/T (0.138)	*NCALD*	Language	0.026 (0.010)	7.77 × 10^–3^	0.002 (0.001)	1.39 × 10^–1^	1.46 × 10^–2^	4.69 × 10^–7^	7.64 × 10^–5^	**1.23 × 10**^**–9**^
					Memory	-0.026 (0.011)	1.65 × 10^–2^	-0.003 (0.001)	1.01 × 10^–2^	9.76 × 10^–3^			
9	10155013	rs145989094	A/T (0.013)	*PTPRD*	Exec Function	-0.105 (0.032)	1.25 × 10^–3^	-0.011 (0.003)	3.18 × 10^–4^	9.49 × 10^–5^	1.09 × 10^–4^	1.19 × 10^–5^	**3.85 × 10**^**–8**^
					Memory	-0.131 (0.034)	1.62 × 10^–4^	-0.015 (0.003)	2.68 × 10^–5^	1.02 × 10^–5^			
9	10155013	rs145989094	A/T (0.013)	*PTPRD*	Language	-0.154 (0.031)	8.58 × 10^–7^	-0.010 (0.003)	4.26 × 10^–3^	1.15 × 10^–6^	1.56 × 10^–6^	1.71 × 10^–4^	**8.34 × 10**^**–9**^
					Memory	-0.131 (0.034)	1.62 × 10^–4^	-0.015 (0.003)	2.68 × 10^–5^	1.02 × 10^–5^			
16	83947805	rs12447050	C/T (0.391)	*OSGIN1* *MLYCD*	Exec Function	0.024 (0.007)	1.02 × 10^–3^	0.003 (0.001)	8.06 × 10^–5^	1.16 × 10^–5^	9.49 × 10^–5^	1.46 × 10^–5^	**4.09 × 10**^**–8**^
					Memory	0.029 (0.008)	1.71 × 10^–4^	0.003 (0.001)	2.00 × 10^–4^	3.00 × 10^–5^			

^a^*A1* Minor allele, *A2* Reference allele, *MAF* Minor allele frequency

**Fig. 2:**
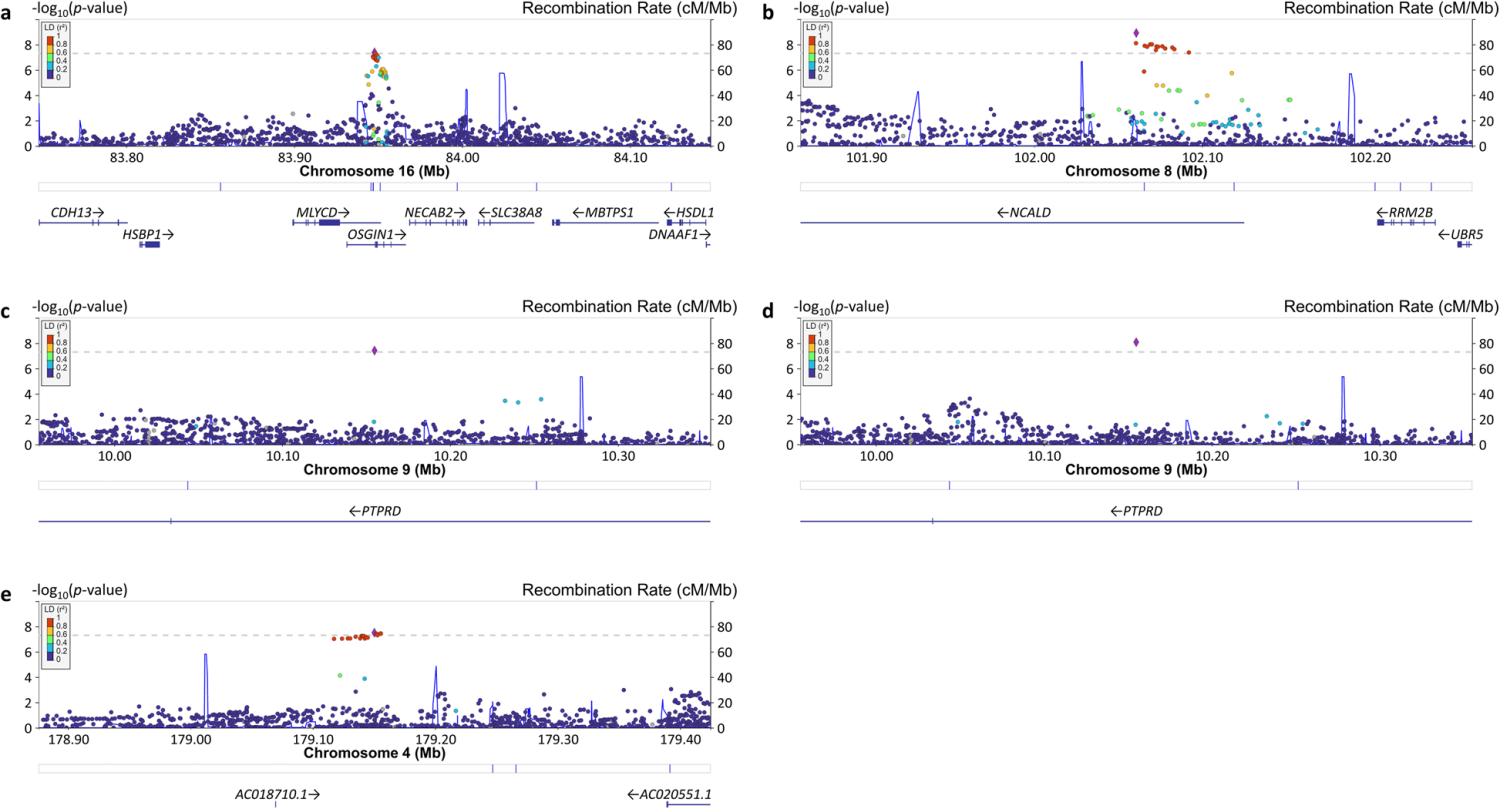
Locus Zoom plots showing genome-wide significant pleiotropy for SNPs in the regions of novel loci. The SNP with the lowest *p*-value at each locus is indicated with a purple diamond. Computed estimates of linkage disequilibrium (*r*^2^) of SNPs in the region with top-ranked SNP are color-coded according to the key. Vertical blue lines indicate locations of high recombination rates. Locations of genes in the region are shown below the diagram. **a** Association of rs12447050 with executive function and memory in the community-based cohorts. **b** Association of rs56162098 with language and memory in the community-based cohorts. **c** Association of rs145989094 with executive function and memory in the community-based cohorts. **d** Association of rs145989094 with language and memory in the community-based cohorts. **e** Association of rs73005629 with language and memory in the clinic-based cohorts

In the clinic-based cohorts, there was GWS pleiotropy for language and memory with rs73005629 (*P*_Joint_ = 3.12 × 10^–8^) located in an intergenic region on chromosome 4 (Table 3, Fig. 2). The joint effect of rs73005629 on language (*P*_Joint_ = 4.66 × 10^–7^) and memory (*P*_Joint_ = 1.47 × 10^–7^) was equally attributable to its main and interaction effects. There was no evidence of pleiotropy for rs73005629 in the community-based cohorts. Conversely, significant pleiotropy for the same domain pair was observed with *NCALD* SNP rs56162098 (*P*_Joint_ = 1.23 × 10^–9^) and *PTPRD* SNP rs145989094 (*P*_Joint_ = 8.34 × 10^–9^) in the community-based cohorts (Table 3). The association with rs56162098 was not evident in the clinic-based cohorts but was supported by the association with neighboring variants (Fig. 2). The same *PTPRD* SNP was also pleiotropic for executive function and memory (*P*_Joint_ = 3.85 × 10^–8^), but this association is not supported by findings in the clinic-based cohorts (Table 3) or neighboring SNPs (Fig. 2).

We also identified significant pleiotropy in the community-based cohorts for executive function and memory with rs12447050, located 5.5 kb upstream from *OSGIN1* (*P*_Joint_ = 4.09 × 10^–8^). This association was comparably supported by each domain and the SNP’s main effect and interaction with age (Table 3), as well as by neighboring SNPs (Fig. 2). There was no evidence of association with the individual domains or in the pleiotropy model in the clinic-based cohorts. However, the magnitude of effect for rs12447050 and its interaction with age in each domain, as well as the significance levels for the main, interaction, and joint pleiotropy tests in the community-based cohorts and the total sample, were nearly identical (Table 3).

Highly suggestive pleiotropy was observed in the community-based cohorts with two SNPs (rs7081658 and rs7070729) located in the *USP6NL*/*ECHDC3* region, an established AD risk locus, for executive function and language (*P*_Joint_ = 3.54 × 10^–7^ and *P*_Joint_ = 8.76 × 10^–8^, respectively) and for executive function and memory (*P*_Joint_ = 2.11 × 10^–7^ and *P*_Joint_ = 5.09 × 10^–8^, respectively); rs7070729 was also pleiotropic for language and memory (*P*_Joint_ = 7.76 × 10^–7^) (Table S4). There was also suggestive pleiotropy for executive function and language with two SNPs in the AD risk locus *WWOX* (rs13329990, *P*_Joint_ = 8.45 × 10^–7^; rs11862902, *P*_Joint_ = 9.60 × 10^–7^).

### Pathways involved in neuronal development or signaling, vascular and endocrine systems are related to cognitive domain performance

A total of 28 canonical pathways were significantly enriched for loci associated with pleiotropy for paired domains (Table 4), noting that none of these pathways were specific to the clinic-based cohorts, and no significant pathways were identified in analyses seeded with top-ranked genes in the GWAS for individual cognitive domains. The evidence for approximately 60% (17/28) of these pathways was derived from analyses of the community-based cohorts only. The top-ranked pathway, synaptogenesis signaling, was significantly enriched for genes that emerged from pleiotropy analysis for all three pairs of cognitive domains. Several pathways are related to neuronal development or signaling (*e*.*g*., synaptogenesis signaling, synaptic long-term depression, endocannabinoid neuronal synapse, netrin signaling, GABA and glutamate receptor signaling, and calcium signaling), AD-associated vascular risk factors (*e*.*g*., type II diabetes and maturity onset diabetes of young signaling, insulin secretion signaling, dilated cardiomyopathy and cardiac hypertrophy signaling, and nitric oxide signaling in the cardiovascular system), and the endocrine system (*e*.*g*., G protein-coupled receptor-mediated nutrient sensing in enteroendocrine cells, insulin, corticotropin-releasing hormone, gonadotropin-releasing hormone, androgen, and oxytocin signaling). Details for suggestive pathways (FDR-adjusted *P* < 0.05) and the number of seed genes selected from each GWAS and pleiotropy analysis are summarized in Tables S5 and S6, respectively.

**Table 4:** Canonical pathways significantly enriched for top-ranked GWAS genes

**Pathway**	**Sample**	**GWAS Outcome**	**Number of genes**	***P***_**FDR**_^**1**^
Synaptogenesis signaling	Community	L / M	334	4.07 × 10^–6^
		EF / L	191	1.62 × 10^–4^
		EF / M	286	6.61 × 10^–4^
Opioid signaling	Community	EF / L	191	8.13 × 10^–6^
Amyotrophic lateral sclerosis signaling	All	EF / L	211	2.14 × 10^–5^
Maturity onset diabetes of young signaling	All	EF / L	211	2.14 × 10^–5^
Corticotropin releasing hormone signaling	Community	EF / L	191	3.39 × 10^–5^
Dilated cardiomyopathy signaling	Community	EF / L	191	3.39 × 10^–5^
Netrin signaling	Community	EF / L	191	3.39 × 10^–5^
Neuronal nitric oxide synthase signaling in skeletal muscle cells	Community	EF / L	191	3.39 × 10^–5^
Circadian rhythm signaling	Community	EF / L	191	5.62 × 10^–5^
Insulin secretion signaling	Community	EF / L	191	5.62 × 10^–5^
White adipose tissue browning	Community	EF / L	191	1.05 × 10^–4^
Synaptic long-term depression	Community	EF / L	191	1.62 × 10^–4^
Nitric oxide signaling in the cardiovascular system	All	EF / L	211	1.74 × 10^–4^
GPCR-mediated nutrient sensing in enteroendocrine cells	Community	EF / L	191	2.63 × 10^–4^
Protein kinase A signaling	Community	EF / L	191	2.63 × 10^–4^
Hepatic fibrosis signaling	Community	EF / L	191	3.24 × 10^–4^
GABA receptor signaling	Community	EF / L	191	3.98 × 10^–4^
Endocannabinoid neuronal synapse	All	EF / L	211	4.68 × 10^–4^
Type II diabetes mellitus signaling	All	EF / L	211	4.68 × 10^–4^
GNRH signaling	Community	EF / L	191	5.25 × 10^–4^
Cardiac hypertrophy signaling (enhanced)	Community	EF / L	191	6.92 × 10^–4^
Gustation pathway	Community	EF / L	191	6.92 × 10^–4^
Androgen signaling	All	EF / L	211	7.94 × 10^–4^
Calcium signaling	All	EF / L	211	7.94 × 10^–4^
Oxytocin signaling	All	EF / L	211	7.94 × 10^–4^
G beta gamma signaling	All	EF / L	211	8.32 × 10^–4^
AMPK signaling	All	EF / M	210	9.55 × 10^–4^
Glutamate receptor signaling	All	EF / M	210	9.55 × 10^–4^

*All* Total sample, *Community* Community-based cohorts, *EF* Executive function, *L* Language, *M* Memory

^1^*P*-values were adjusted for a false discovery rate and 18 separate pathway analyses (threshold *P* < 0.001)

## Discussion

Genome-wide scans for performance measures in three cognitive domains in two large clinically ascertained and three community-based cohorts revealed GWS associations with four well-established AD loci (*BIN1*, *CR1*, *MS4A6A*, and *APOE*) and eight loci not previously genetically linked to AD or cognitive decline (*ULK2*, *CDK14*, *PURG*, *LINC02712*, *LOC107984373*, *NCALD*, *PTPRD*, and *OSGIN1*), as well as with *GRN* which has been associated with AD and several other dementing illnesses [7, 10, 78–80]. These findings were based on analyses that leveraged data obtained from one or more cognitive examinations, considered cognitive performance changes over time, and examined genetic effects on individual or pairs of domains. In comparison to previous GWAS of cognitive performance, which were limited to the availability of data for particular NP tests and focused primarily on clinic-based or community-based samples [26–29], our study utilized harmonized measures that enabled pooling data obtained using multiple NP protocols and considered associations that may be common or unique to differentially ascertained samples.

To our knowledge, this is the first genome-wide pleiotropy study using harmonized cognitive domain scores. Compared to previous conventional GWAS or pleiotropy studies of individual cognitive traits [26–29], our genetic analysis of harmonized cognitive scores allows combining results from studies using different NP protocols and permits greater opportunities for replication and meta-analyses. This approach has been successfully used in a variety of studies of cognitive aging [81–84]. A recent study of five preclinical AD cohorts conducted a factor analysis on three domains—general cognitive performance, episodic memory, and executive function—and established a common algorithm for classifying MCI progression across the heterogeneously evaluated samples [85]. Cognitive factor scores derived in an identical fashion as those used in this study have been utilized for a variety of investigations of AD subgroups [81], which linked cognition to imaging [83], neuropathology [84], and genetics [15]. Similarly, they have been used in genetic studies of cognitive resilience to AD [82, 86].

We identified three novel loci that have functional relevance to processes implicated in AD. Dysfunction of the protein encoded by *ULK2*, unc51 like autophagy activating kinase 2, has been suggested to cause multiple diseases. *ULK2* SNPs have been associated with schizophrenia [87], and a *ULK2* circular RNA is expressed more than tenfold in a vascular dementia rat model [88]. Lee and colleagues recently demonstrated that amyloid-*β* 42 oligomer-mediated loss of excitatory synapses in cortical neurons and hippocampal CA1 neurons requires *AMPK*-mediated activation of *ULK2*-dependent mitophagy [89]. *PTPRD*, protein tyrosine phosphatase receptor type D, was previously reported to be associated with AD susceptibility [90]. A recent study identified a significant association of *PTPRD* with the accumulation of neurofibrillary tangles that was independent of amyloid-*β* pathology [20]. *NCALD* encodes a member of the neuronal calcium sensor family of calcium-binding proteins, which mediates signal transduction in response to calcium in neurons. *NCALD* is downregulated in the AD brain and may play a protective role in hippocampal CA1 and CA3 regions [91, 92]. This observation is consistent with a finding from a study of differentially expressed proteins in rats fed a high-fat diet suggesting that the memory-impairing effects of diet-induced obesity might potentially be mediated by down-regulated *NCALD* within the hippocampus [93].

We also identified a GWS signal in *GRN*, the gene that encodes the anti-inflammatory and neurotrophic factor progranulin (PGRN) [94]. *GRN* mutations are a well-established cause of frontotemporal lobar degeneration (FTLD). More than 60 disease-causing *GRN* mutations have been identified, accounting for 20% to 25% of familial FTLD cases and about 10% of all FTLD cases [95]. The most significantly associated *GRN* SNP in our study, rs5848, was found in the 3’-untranslated region, which is predicted to be a microRNA binding site. Rs5848 is the *GRN* variant most frequently associated with FTLD and is associated with a reduction in PGRN in plasma and cerebrospinal fluid [96, 97]. In addition to FTLD, several studies have shown an association between clinical AD and the rs5848 *T* allele, which we found to be linked to lower memory performance in both clinic- and community-based cohorts [98]. A recent large GWAS meta-analysis found a GWS association of AD risk with rs5848-*T* [10]. A recent study examining neuropathological AD correlates showed that rs5848 *T* allele carriers had a higher frequency of hippocampal sclerosis and TDP-43 deposits, significantly increased tau pathology burden, but showed no specific association with *β*-amyloid load or AD neuropathological diagnosis [99]. Interestingly, our finding was exclusive to the memory domain, which is affected in early stages of AD, but also commonly affected in hippocampal sclerosis and limbic-predominant age-related TDP-43 encephalopathy (LATE) [100, 101]. Effects were driven by both the SNP’s main and SNP × age interaction effects. This finding provides additional evidence that variation in *GRN* may be related to neurodegeneration more broadly and that restoring PGRN levels may be an effective way to prevent and treat dementia [102].

Highly suggestive pleiotropy (*P* < 1 × 10^–7^) was also found with other established AD risk loci, including *USP6NL*/*ECHDC3* for all three paired cognitive domains and *WWOX* for executive function and language. *ECHDC3*, enoyl-CoA hydratase domain containing 3, was previously reported to be associated with AD [7, 10]. *ADCY2*, adenylate cyclase 2, was reported to be associated with AD-related changes in hippocampal gene expression [103, 104], as well as AD-associated structural changes detected by brain imaging [105]. A recent GWAS reported the association of *DAPK2*, death associated protein kinase 2, with amyloid deposition in the brain [106], a finding consistent with studies showing that *DAPK1* promotes *APP* phosphorylation and amyloidogenic processing [107]. *PLXDC2*, plexin domain containing 2, is upregulated with increasing *β*-amyloid plaque load or Braak stages [108].

There are no established links of *OSGIN1*, *CDK14*, and *PURG* to AD. The product encoded by *OSGIN1* is an oxidative stress response protein that regulates cell death and appears to be a key regulator of both inflammatory and anti-inflammatory molecules [109, 110]. *CDK14* encodes a protein kinase whose expression is more than two-fold higher in the brain than in any other tissue. However, it has been linked to cancer in various tissues, primarily outside of the brain. Although the function of *PURG* is unknown, a SNP in this gene showed significant associations in GWAS of cognitive performance and intelligence [111, 112]. The biological significance of the pleiotropic association of memory and language with a chromosome 4 variant located about 170 kb from *LOC107984373*, which encodes a long non-protein coding RNA, is also puzzling at this time.

Bioinformatic analyses of the top-ranked genes emerging from GWAS implicated several biological pathways related to neuronal development and signaling, AD-associated vascular risk factors, and endocrine pathways. Notably, all of the significant pathways were identified from analyses of findings from the pleiotropy GWAS analyses, especially those supported by the community-based cohorts. Because pathways were constructed using information from well-established metabolic and cell signaling pathways, they tend to reflect more common or shared mechanisms rather than particular or trait-specific mechanisms. Therefore, pleiotropic loci affecting multiple cognitive domains may be more suitable as seed genes for canonical pathways than loci associated with a single domain. Indeed, in the analyses of community-based cohorts, the numbers of seed genes from pleiotropy GWAS were 1.5–1.7 times larger than those from GWAS of individual cognitive domains (Table S6). Considering that AD pathology results in progressive dysfunction in several cognitive domains over time, the majority of our findings, which emerged from analyses of the community-based rather than the clinic-based cohorts, may represent pathways underlying cognitive processes related to AD progression rather than AD risk.

Interestingly, associations with several well-established AD loci, including *BIN1*, *CR1*, and *MS4A6A*, were observed only in the clinic-based cohorts. Lack of replication in the community-based cohorts might be due to the relative paucity of AD cases and the higher likelihood of mixed pathologies. Conversely, the associations with the known AD locus *USP6NL*/*ECHDC3* and novel loci, including *ULK2*, *NCALD*, *PTPRD*, *ADCY2*, and *OSGIN1*, were observed only in the community-based cohorts. Lack of replication in the clinic-based cohorts may indicate that these loci are associated with normal age-related cognitive changes rather than an AD process. However, this explanation seems less likely given their previous association with AD risk (*USP6NL*/*ECHDC3*) or functional relevance to processes implicated in AD and/or their association with other AD-related endophenotypes (*NCALD*, *ULK2* and *PTPRD*). Alternatively, community-based cohort-specific findings may indicate that the effects of these genes are age-dependent or detectable when tracked over time. This idea is supported by the observation of the highly significant SNP × age interaction term for these loci, which for *ULK2*, *PTPRD*, *ADCY2*, and *OSGIN1* were responsible for the significant joint effect to a much greater extent than the SNP’s main effect.

We employed a joint test that combines the main genetic effects and SNP × age interaction together to increase our power to detect genetic associations. Nonetheless, interpreting a joint test can be challenging, requiring examination of effect estimates for both the main and interaction terms. The contribution of the SNP’s main effect to the joint association for some findings, including the well-established AD loci and several novel ones (*e*.*g*., *ULK2*, *CDK14*, *PURG*, *LINC02712*, and *GRN*), was much stronger than its interaction with age. This may reflect that these loci are associated with the development rather than the progression of AD. This aligns with the fact that these loci were initially identified using a case-control design. In contrast, particularly for the more novel loci, the contribution of the SNP × age interaction to the joint association was stronger than the main effect. This may reflect that these loci are associated with the progression rather than the development of AD and could explain why they have not been identified previously, as few genetic studies of AD have utilized a longitudinal design.

Of note, all of the GWS pleiotropic associations and half of the GWS single-domain associations involved the memory domain. This is not surprising as prominent memory impairment is the most common cognitive feature in AD. Nonetheless, prominent impairment in other cognitive domains occurs in a reasonable number of AD cases. Those specific loci were implicated for particular cognitive domains may provide syndrome-specific therapeutic targets with an eye toward a precision medicine approach to AD.

Our results also highlight that the biology underlying cognitive performance in older individuals is complex and likely a function of multiple processes including lifelong ability, neurodegeneration and resilience to neurodegeneration. Genetic architecture may be influencing cognition through each of these processes. Without a measure of underlying pathology, disentangling the mechanism by which genes are affecting cognition is difficult. This point applies to both the current study and the large AD GWAS in which most participants received a diagnosis based only upon assessment of cognition in life. Our finding that AD risk is strongly genetically correlated with the factor score for memory but not executive function or language might provide some insight into these processes. GWAS findings for the individual cognitive domains showing that memory was associated only with established AD risk genes. However, all of the novel associations identified in the pleiotropy analysis included memory as part of the paired outcome. These genetic association patterns might argue that our phenotypes for executive function and language could reflect decline from AD (rather than development of AD), underlying cognitive ability and/or cognitive resilience. Future studies that utilize both measures of cognition and underlying pathology will be needed to better disentangle the genetic architecture underlying these different processes that influence cognition.

This study has several limitations. Although we included several large cohorts whose cognitive test data were co-calibrated and harmonized with each other, the sample size was small compared to the previous GWAS of AD risk. In addition, there was a reduction in power for tests of marginal genetic effects or SNP × age interactions because a large portion of the subjects had only one visit (FHS-21.5%, NACC-21.1%, ACT-9.9%, ADNI-13.3%, and ROSMAP-6.0%). The interpretation of our findings based on the joint effect of the SNP and SNP × age interaction is complicated because the identified loci could imply several meanings to cognitive domain functions or AD. Those findings may reflect genetic associations with the development or progression of AD or both, but additional work is needed to address this issue confidently. Further, our model assumes linearity in the cognitive trajectories, but cognitive trajectories at different disease stages may be non-linear. The observed associations with known AD loci provide validation for our modeling approach. Our results are not adjusted for the number of genome-wide scans performed, but the analyses for each cognitive domain and paired cognitive domain are testing separate hypotheses. Correction for analyses conducted separately in the clinic- and community-based cohorts would raise the significance threshold to 2.5 × 10^–8^, which would render associations for *MS4A6A*, *LINC02712*, *GRN*, *LOC107984373, PTPRD*, and *OSGIN1* as borderline GWS. Another concern is the lack of replication which will require the availability of co-calibrated longitudinally obtained cognitive data from independent samples which are informative for AD. Ongoing phenotype harmonization efforts of the Alzheimer’s Disease Genetics Consortium, Alzheimer’s Disease Sequencing Project, and other studies will likely yield the data necessary for replication testing. Because some of the identified loci have no obvious connection to AD or cognition, further research is required to determine their mechanistic pathways. Finally, datasets from other population groups containing cognitive domain factor scores for adequately powered samples will be needed to extend our findings which were derived from non-Hispanic whites only.

## Conclusion

Our results provide some insight into biological pathways underlying processes leading to domain-specific cognitive impairment and AD. The findings may provide a conduit toward a syndrome-specific precision medicine approach to AD. Increasing the number of datasets by harmonizing measures of cognitive performance in other cohorts, as applied in this study, would likely enhance the discovery of additional genetic factors of cognitive decline leading to AD and related dementias.

## Supplementary Information

**Additional file 1: Fig. S1.** Schemes for combining results from GWAS and pleiotropy analyses across datasets. **Fig. S2.** Manhattan and QQ plots for GWAS of individual cognitive domain scores in the total sample. **Fig. S3.** Manhattan and QQ plots for GWAS of individual cognitive domain scores in the clinic-based cohorts. **Fig. S4.** Manhattan and QQ plots for GWAS of individual cognitive domain scores in the community-based cohorts. **Fig. S5.** Locus Zoom plots showing association of SNPs in the *BIN1* region with language. **Fig. S6.** Locus Zoom plots showing association of SNPs in the *BIN1* region with memory. **Fig. S7.** Locus Zoom plots showing association of SNPs in the*CR1* and*MS4A6A* regions with memory in the clinic-based cohorts. **Fig. S8.** Manhattan and QQ plots for pleiotropy GWAS in pairs of cognitive domain scores in the total sample. **Fig. S9.** Manhattan and QQ plots for pleiotropy GWAS in pairs of cognitive domain scores in the clinic-based cohorts. **Fig. S10.** Manhattan and QQ plots for pleiotropy GWAS in pairs of cognitive domain scores in the community-based cohorts. **Fig. S11.** Locus Zoom plots showing pleiotropy of SNPs in the *BIN1* region with language and memory.

**Additional file 2: Supplementary Table 1.** Correlations among cognitive domain scores by cohort. **Supplementary Table 2.** Genetic correlations of cognitive domain scores with general cognitive function and neuropsychiatric disorders. **Supplementary Table 3.** Genome-wide significant and suggestive associations for cognitive domain scores in the total sample, clinic-based cohorts, and community-based cohorts. **Supplementary Table 4.** Genome-wide significant and suggestive pleiotropy for each pair of cognitive domains in the total sample, clinic-based cohorts, and community-based cohorts. **Supplementary Table 5.** Canonical pathways significantly enriched for top-ranked GWAS genes with corrected FDR < 0.05. **Supplementary Table 6.** Number of seed genes from individual GWAS and pleiotropy analyses.

## Data Availability

Genetic and phenotype data for the ACT, ADNI, NACC, and ROSMAP GWAS datasets are available through the National Institute on Aging Genetics of Alzheimer Disease Data Storage site (https://www.niagads.org/). Framingham Heart Study data are available in dbGaP and/or through application to the FHS Research Committee (https://www.framinghamheartstudy.org/fhs-for-researchers/). GWAS summary statistics are available in NIAGADS.

## Abbreviations

ACT: Adult Changes in Thought
AD: Alzheimer’s disease
ADNI: Alzheimer’s Disease Neuroimaging Initiative
FDR: False discovery rate
FHS: Framingham Heart Study
FTLD: Frontotemporal lobar degeneration
GRM: Genetic relationship matrix
GWAS: Genome-wide association study
GWS: Genome-wide significant
HWE: Hardy-Weinberg equilibrium
IBD: Identical by descent
LATE: Limbic-predominant age-related TDP-43 encephalopathy
LD: Linkage disequilibrium
MAF: Minor allele frequency
MAGEE: Mixed‐model association test for gene-environment interactions
MCI: Mild cognitive impairment
MMSE: Mini-mental State Examination
NACC: National Alzheimer’s Coordinating Center
NP: Neuropsychological
PC: Principal component
PRGN: Progranulin
QC: Quality control
ROSMAP: Religious Order Study and Rush Memory Aging Project
SE: Standard error
SNP: Single nucleotide polymorphism
TOPMed: Trans-Omics for Precision Medicine
