# Supplementary material for: A genome-wide search for pleiotropy in more than 100,000 harmonized longitudinal cognitive domain scores
Source: Mol Neurodegener. 2023 Jun 22;18:40. doi: 10.1186/s13024-023-00633-4 (PMC10286470; doi:10.1186/s13024-023-00633-4)
Supplement: Supplementary file 1 — Additional file 1: Fig. S1. Schemes for combining results from GWAS and pleiotropy analyses across datasets. Fig. S2. Manhattan and QQ plots for GWAS of individual cognitive domain scores in the total sample. Fig. S3. Manhattan and QQ plots for GWAS of individual cognitive domain scores in the clinic-based cohorts. Fig. S4. Manhattan and QQ plots for GWAS of individual cognitive domain scores in the community-based cohorts. Fig. S5. Locus Zoom plots showing association of SNPs in the BIN1 region with language. Fig. S6. Locus Zoom plots showing association of SNPs in the BIN1 region with memory. Fig. S7. Locus Zoom plots showing association of SNPs in theCR1 andMS4A6A regions with memory in the clinic-based cohorts. Fig. S8. Manhattan and QQ plots for pleiotropy GWAS in pairs of cognitive domain scores in the total sample. Fig. S9. Manhattan and QQ plots for pleiotropy GWAS in pairs of cognitive domain scores in the clinic-based cohorts. Fig. S10. Manhattan and QQ plots for pleiotropy GWAS in pairs of cognitive domain scores in the community-based cohorts. Fig. S11. Locus Zoom plots showing pleiotropy of SNPs in the BIN1 region with language and memory. [file 13024_2023_633_MOESM1_ESM.docx]

**Fig. S1. Schemes for combining results from GWAS and pleiotropy analyses across datasets**

**(a) Total sample**

**
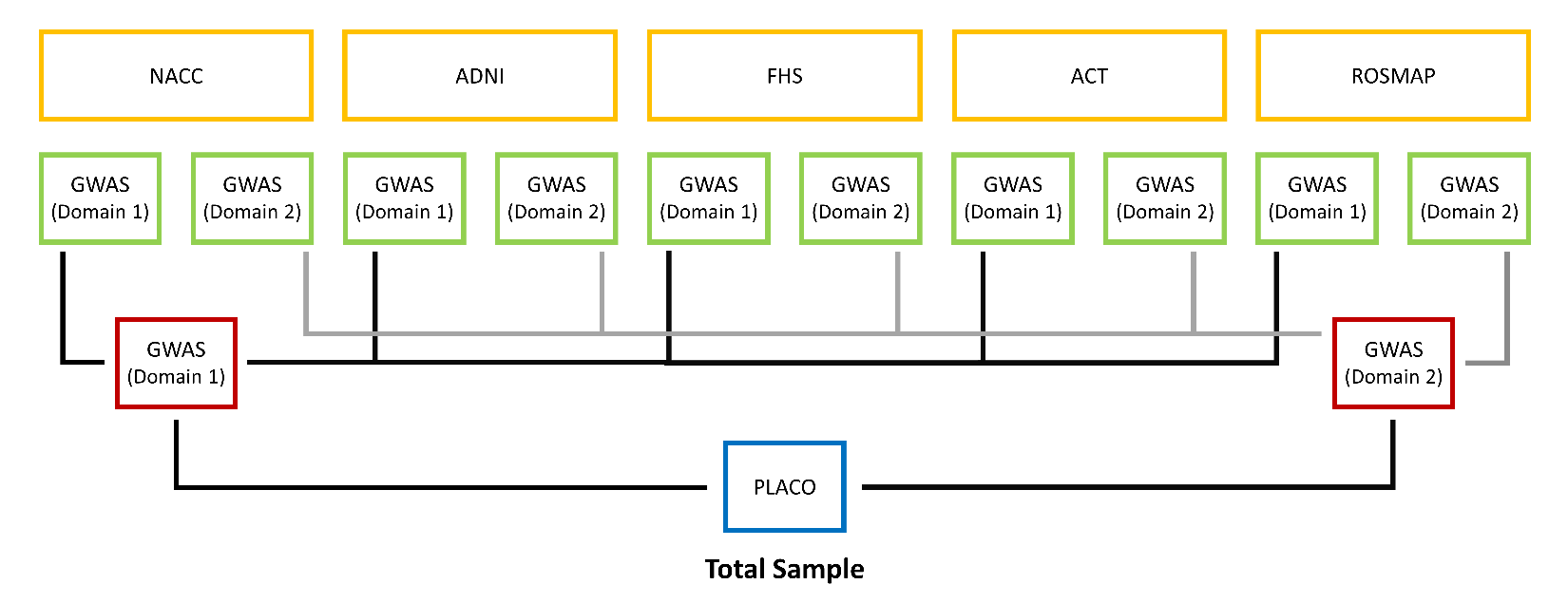
**

**(b) Clinic-based and community-based cohorts**

**
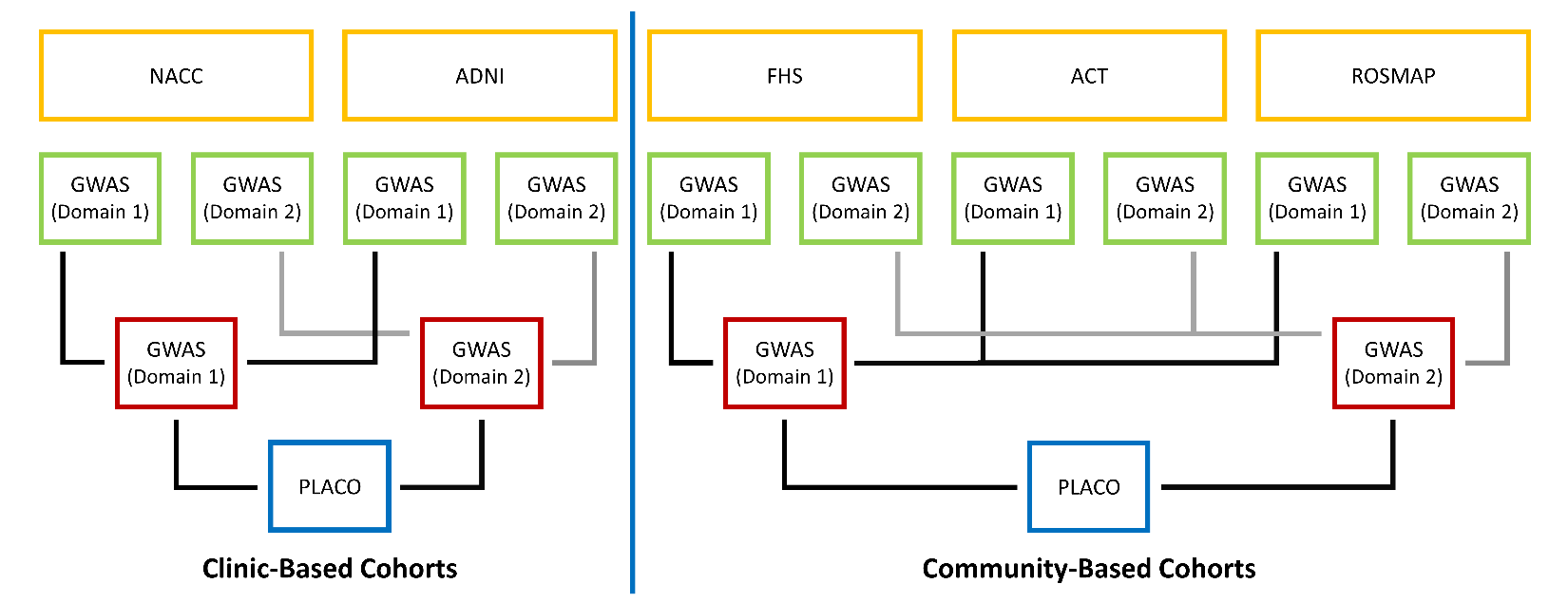
**

(Domain 1, Domain 2): (Executive Function, Language) or (Executive Function, Memory) or (Language, Memory)

**Fig. S2. Manhattan and QQ plots for GWAS of individual cognitive domain scores in the total sample**

**(a) Executive function**


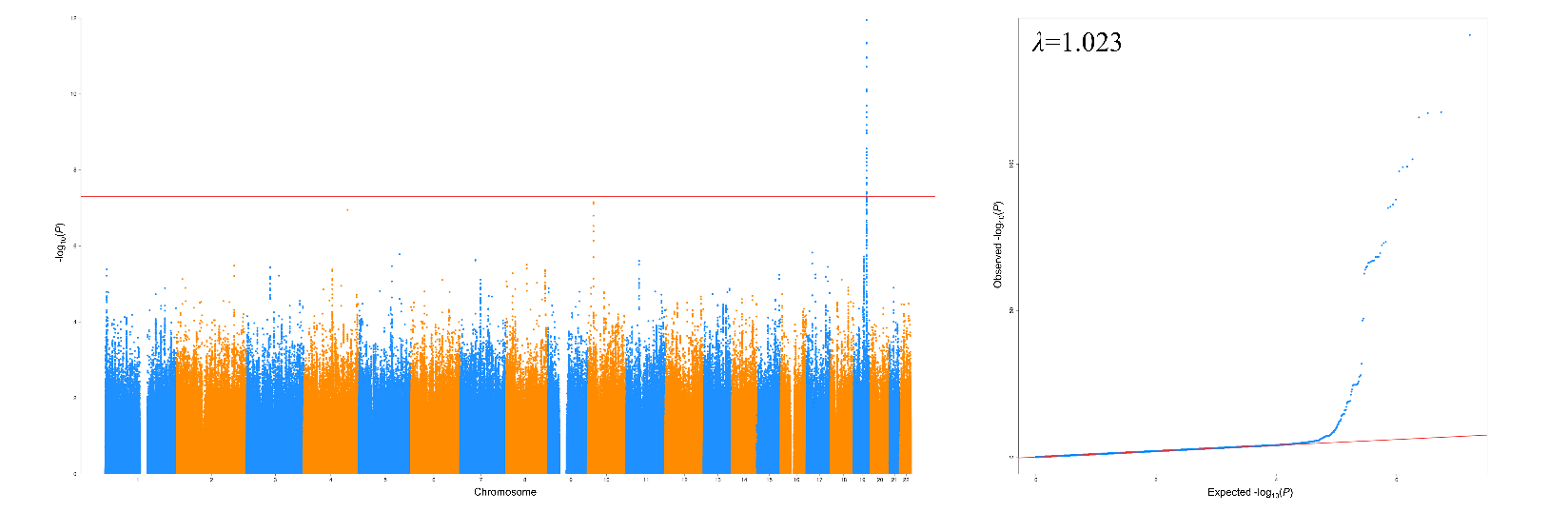


**(b) Language**


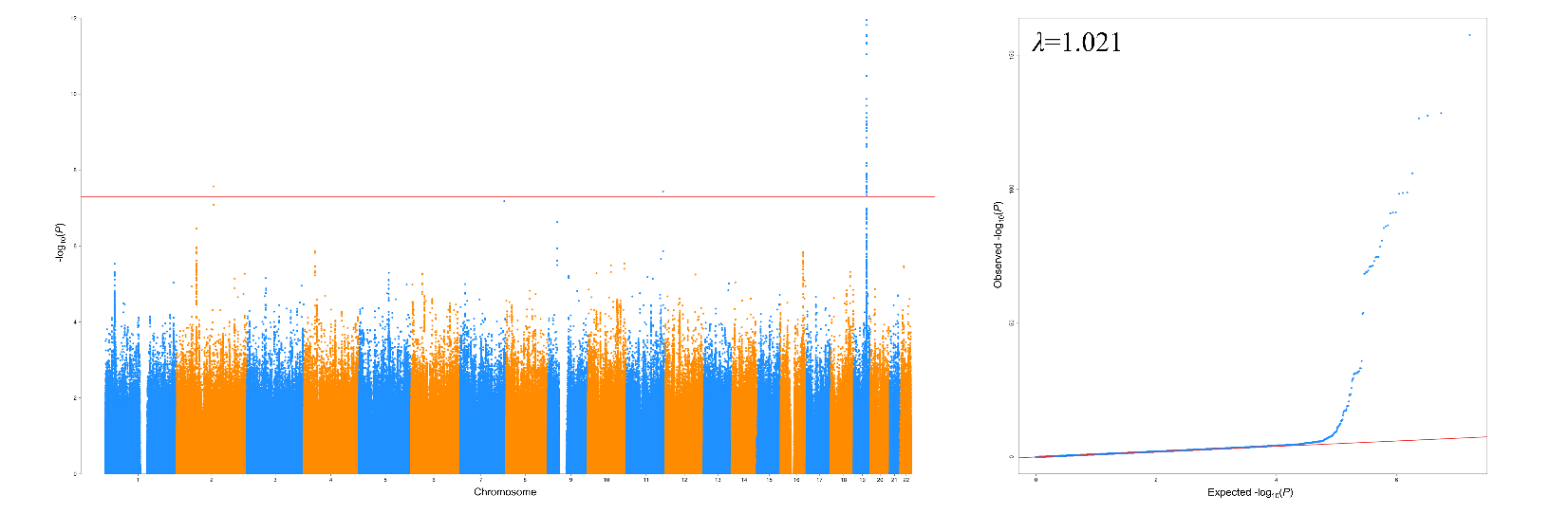


**(c) Memory**


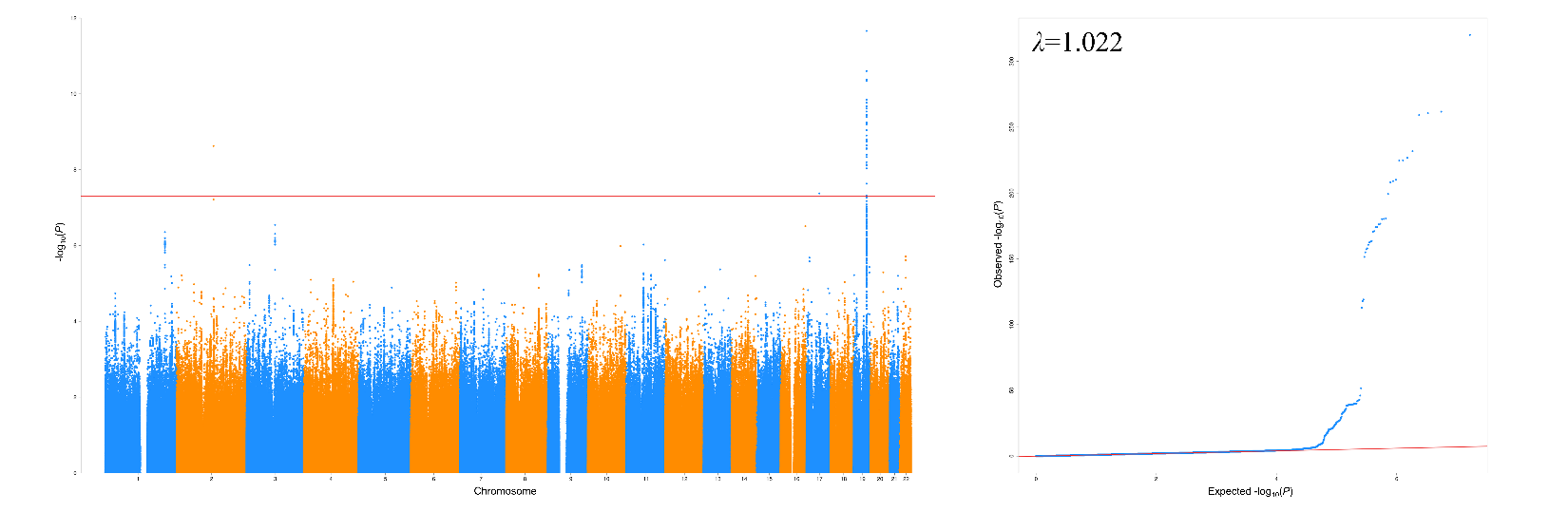


**Fig. S3. Manhattan and QQ plots for GWAS of individual cognitive domain scores in the clinic-based cohorts**

**(a) Executive function**


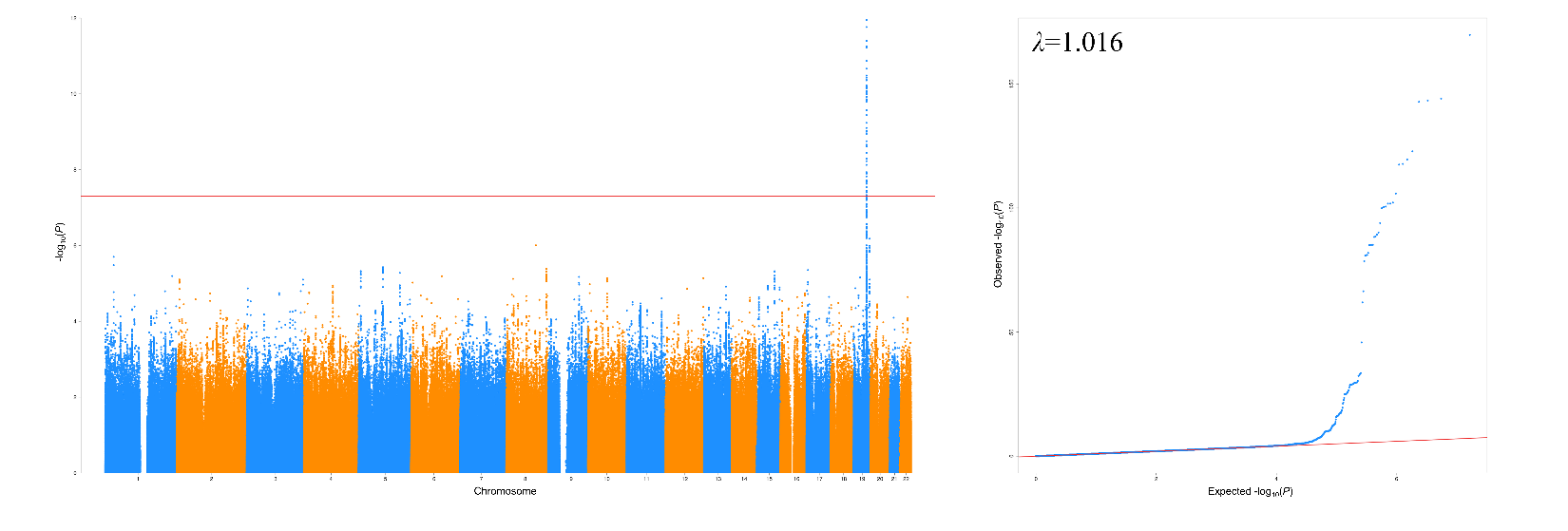


**(b) Language**


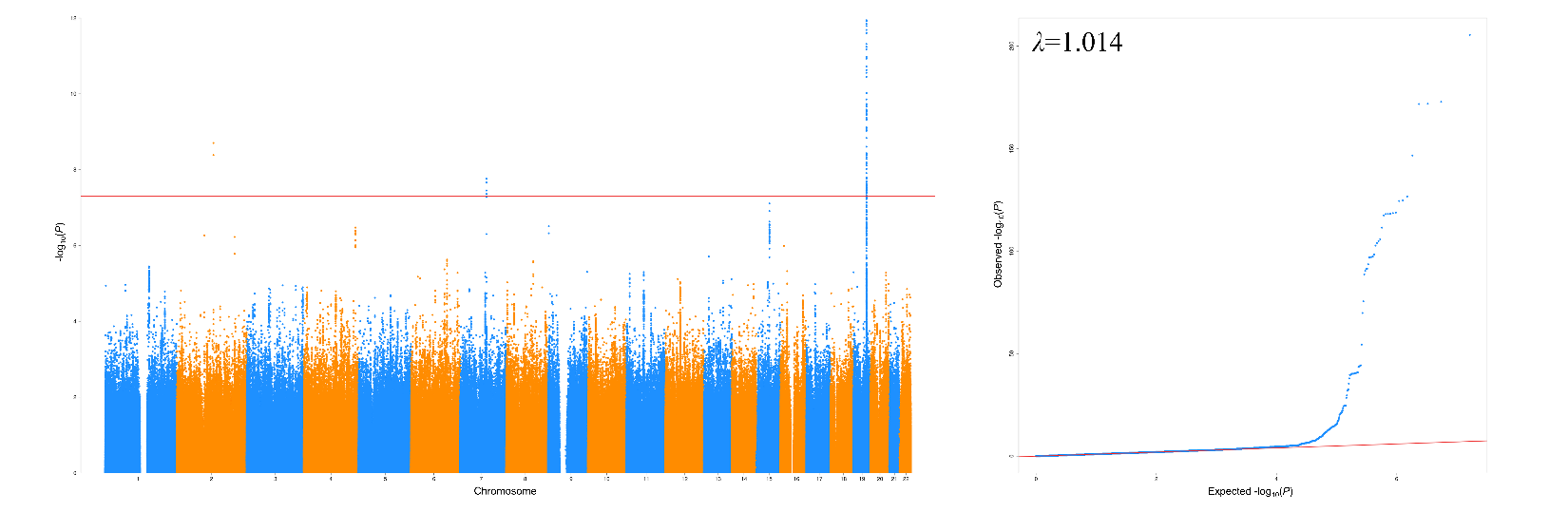


**(c) Memory**


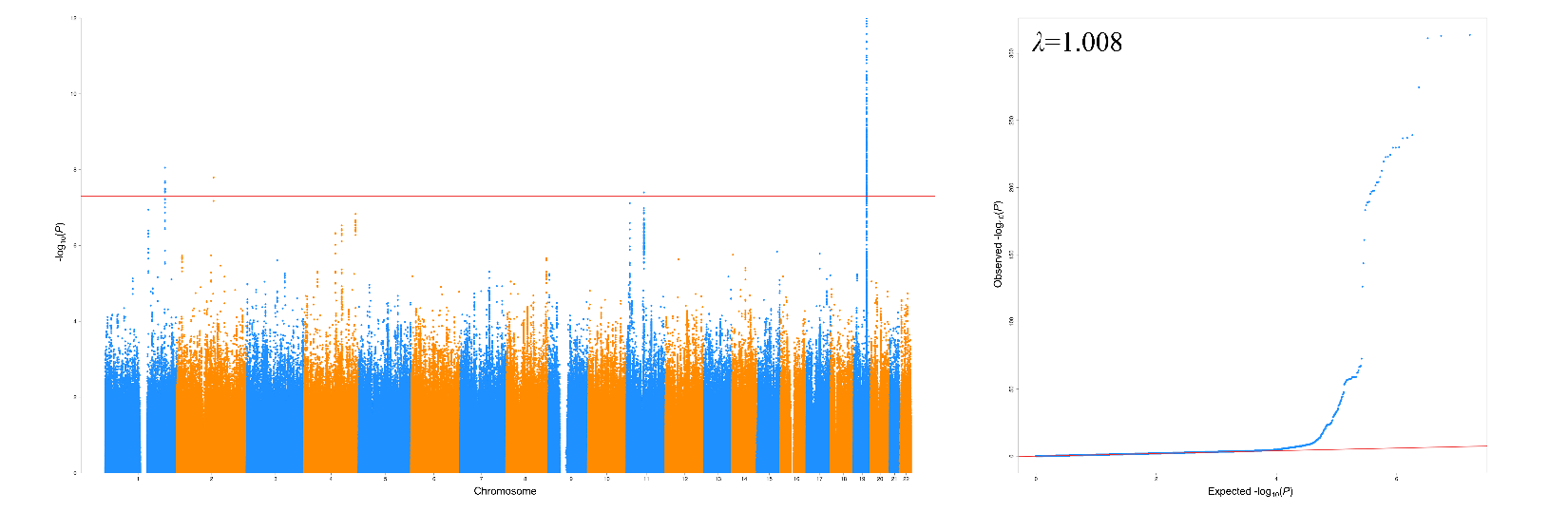


**Fig. S4. Manhattan and QQ plots for GWAS of individual cognitive domain scores in the community-based cohorts**

**(a) Executive function**


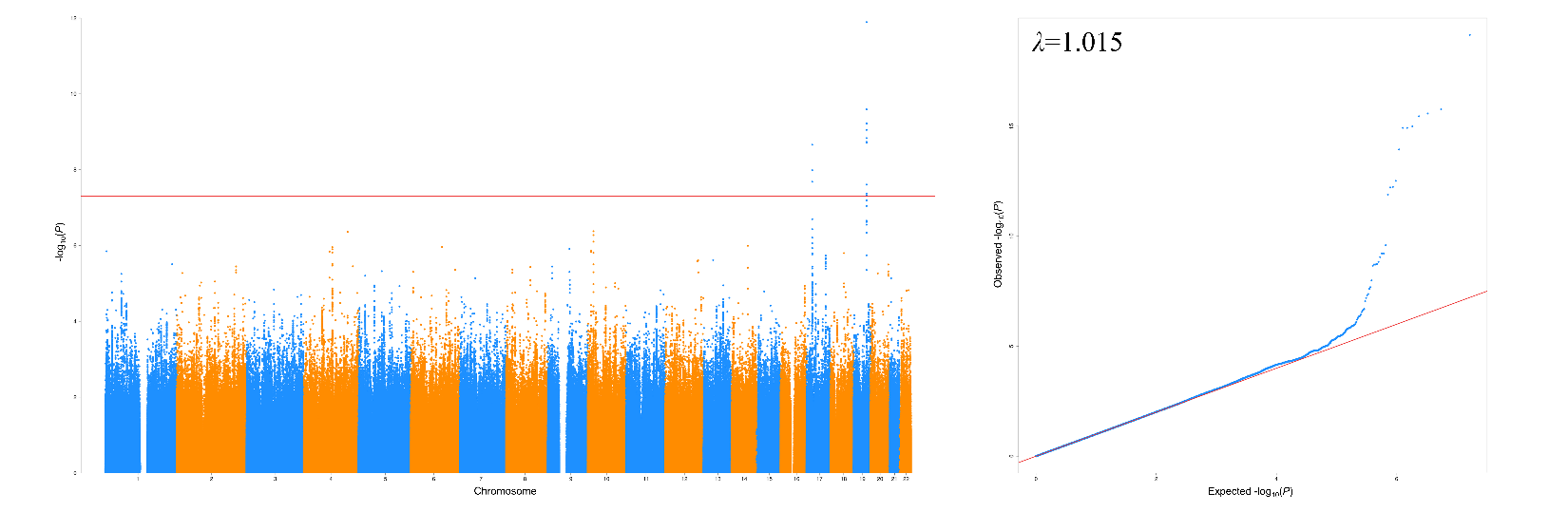


**(b) Language**


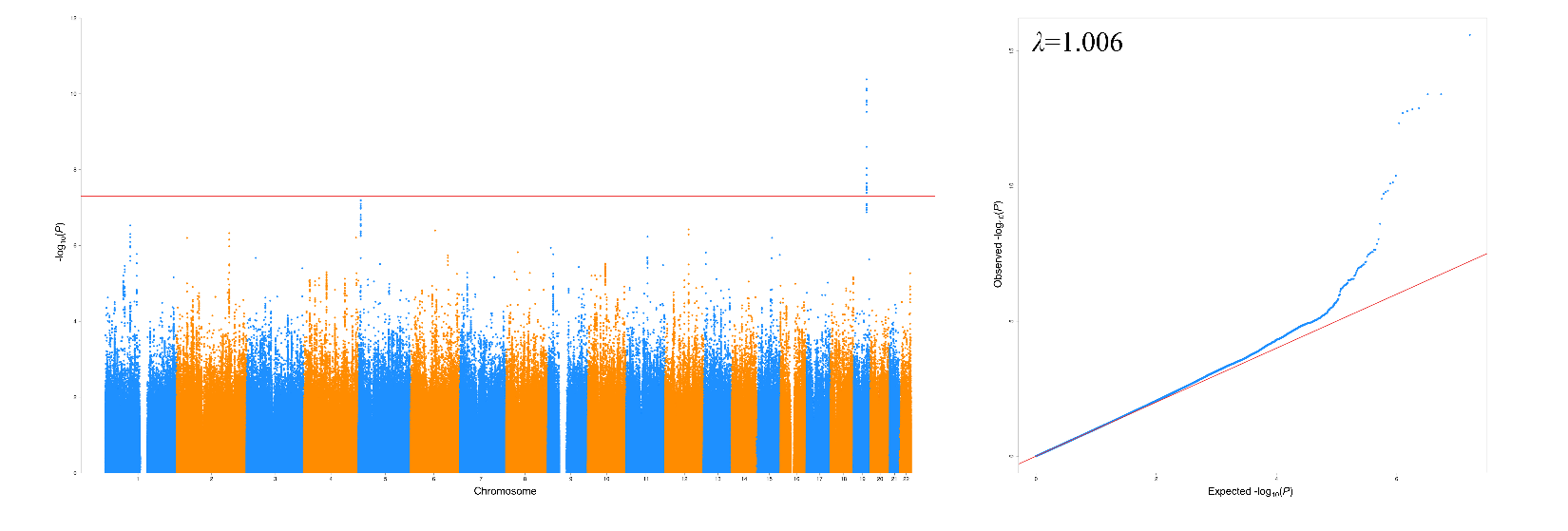


**(c) Memory**


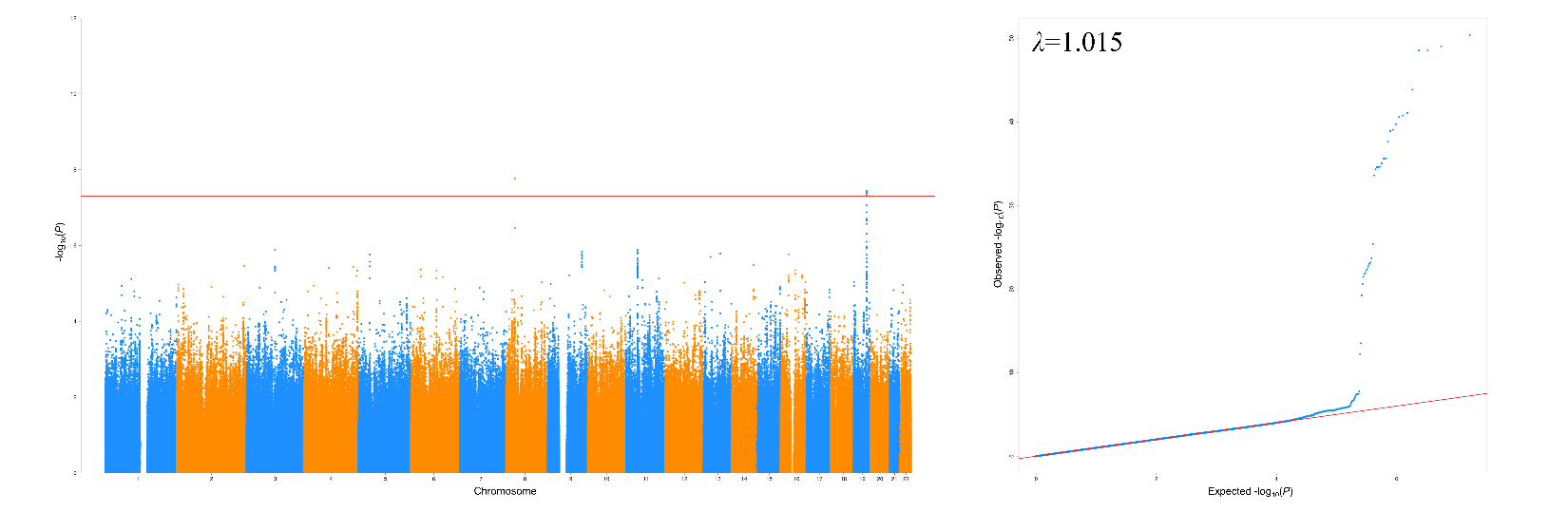


**Fig. S5. Locus Zoom plots showing association of SNPs in the *BIN1* region with language.** The SNP with the lowest *p*-value (rs6733839) is indicated with a purple diamond. Computed estimates of linkage disequilibrium (*r*^2^) of SNPs in the region with rs6733839 are color-coded according to the key. Vertical blue lines indicate locations of high recombination rates. Locations of genes in the region are shown below the diagram.

**(a) Total sample**


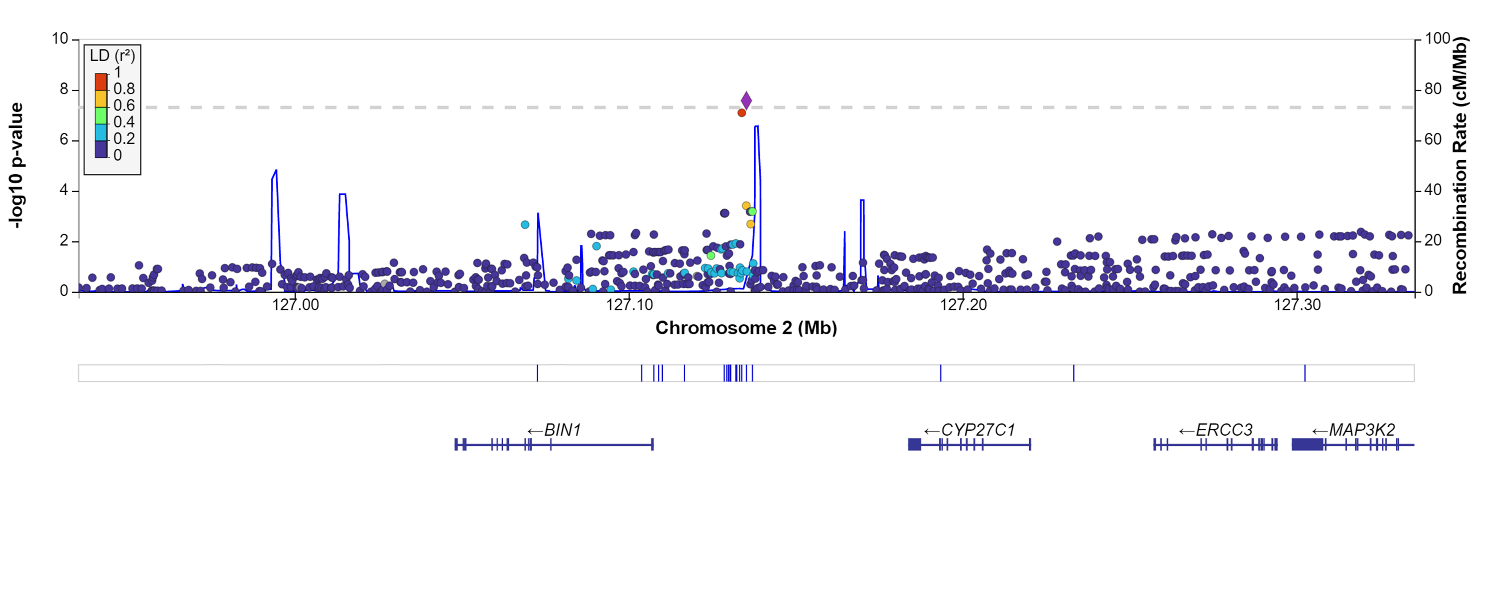


**(b) Clinic-based cohorts**


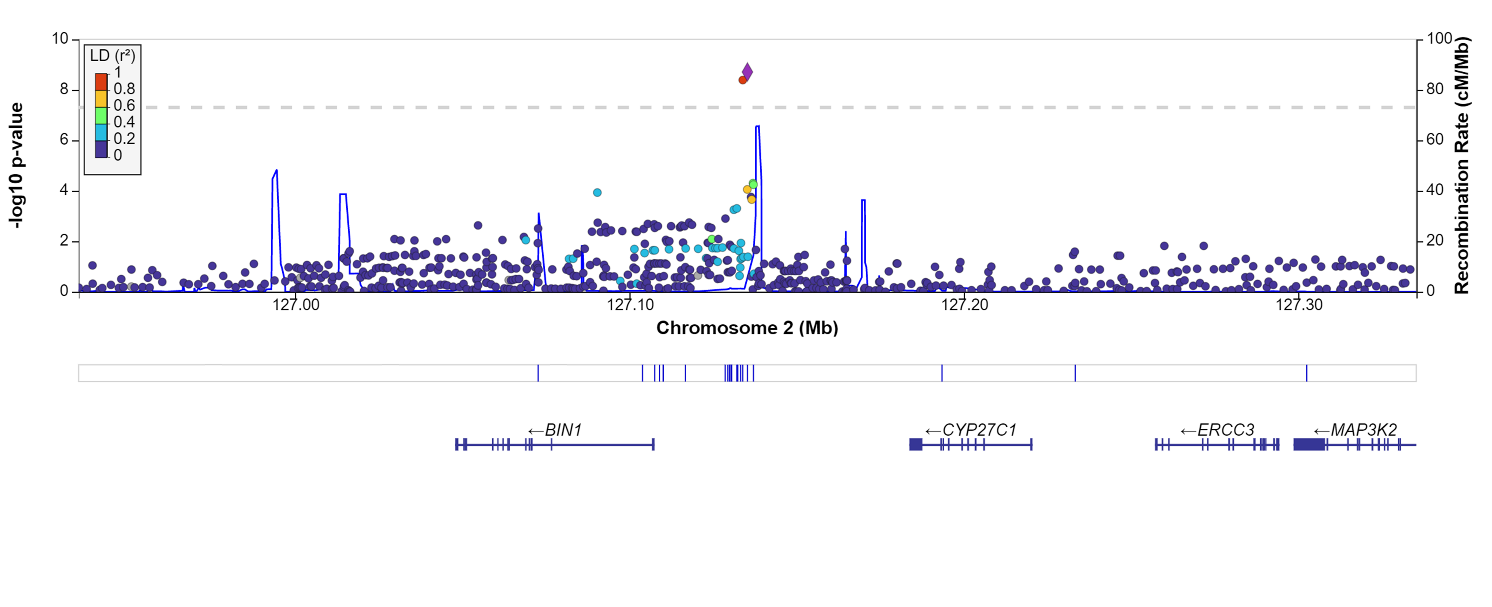


**Fig. S6. Locus Zoom plots showing association of SNPs in the *BIN1* region with memory.** The SNP with the lowest *p*-value (rs6733839) is indicated with a purple diamond. Computed estimates of linkage disequilibrium (*r*^2^) of SNPs in the region with rs6733839 are color-coded according to the key. Vertical blue lines indicate locations of high recombination rates. Locations of genes in the region are shown below the diagram.

**(a) Total sample**


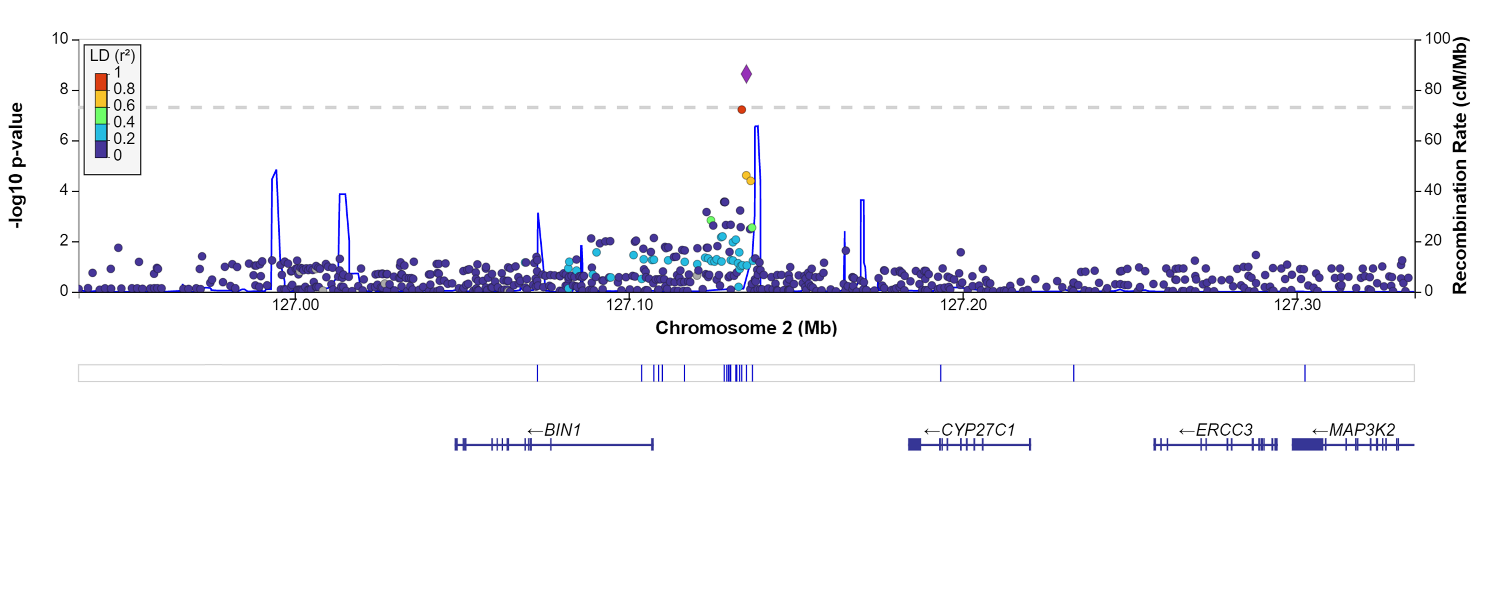


**(b) Clinic-based cohorts**


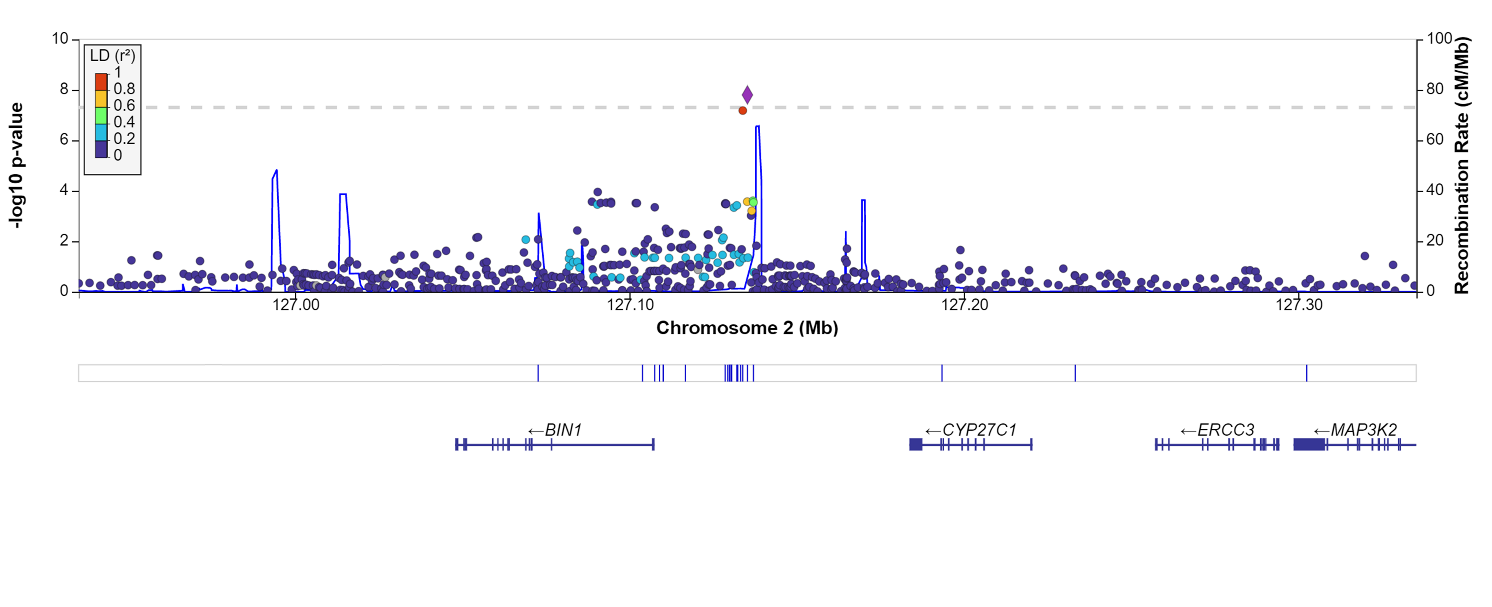


**Fig. S7. Locus Zoom plots showing association of SNPs in the (a) *CR1* and (b) *MS4A6A* regions with memory in the clinic-based cohorts.** The SNP with the lowest *p*-value in each plot (rs1752684 for CR1 and rs7232 for MS4A6A) is indicated with a purple diamond. Computed estimates of linkage disequilibrium (*r*^2^) of SNPs in the region with the top SNP are color-coded according to the key. Vertical blue lines indicate locations of high recombination rates. Locations of genes in the region are shown below the diagram.

**(a) *CR1* region**


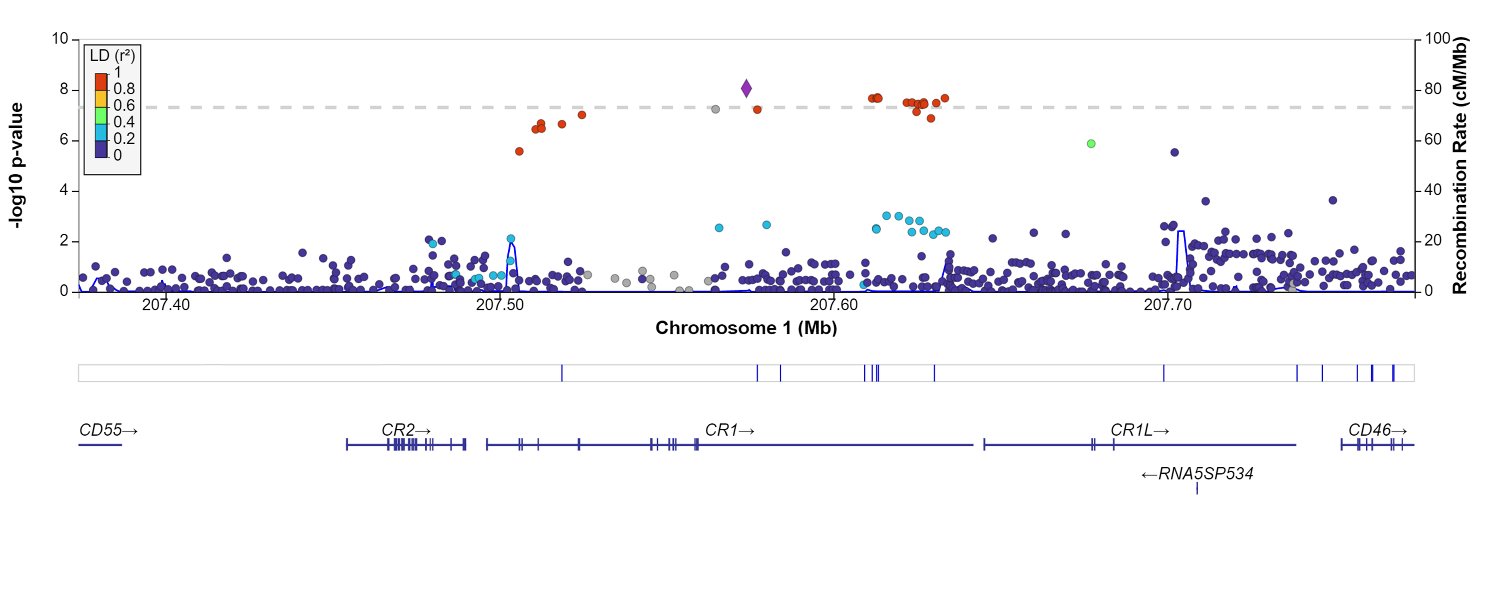


**(b) *MS4A6A* region**

**
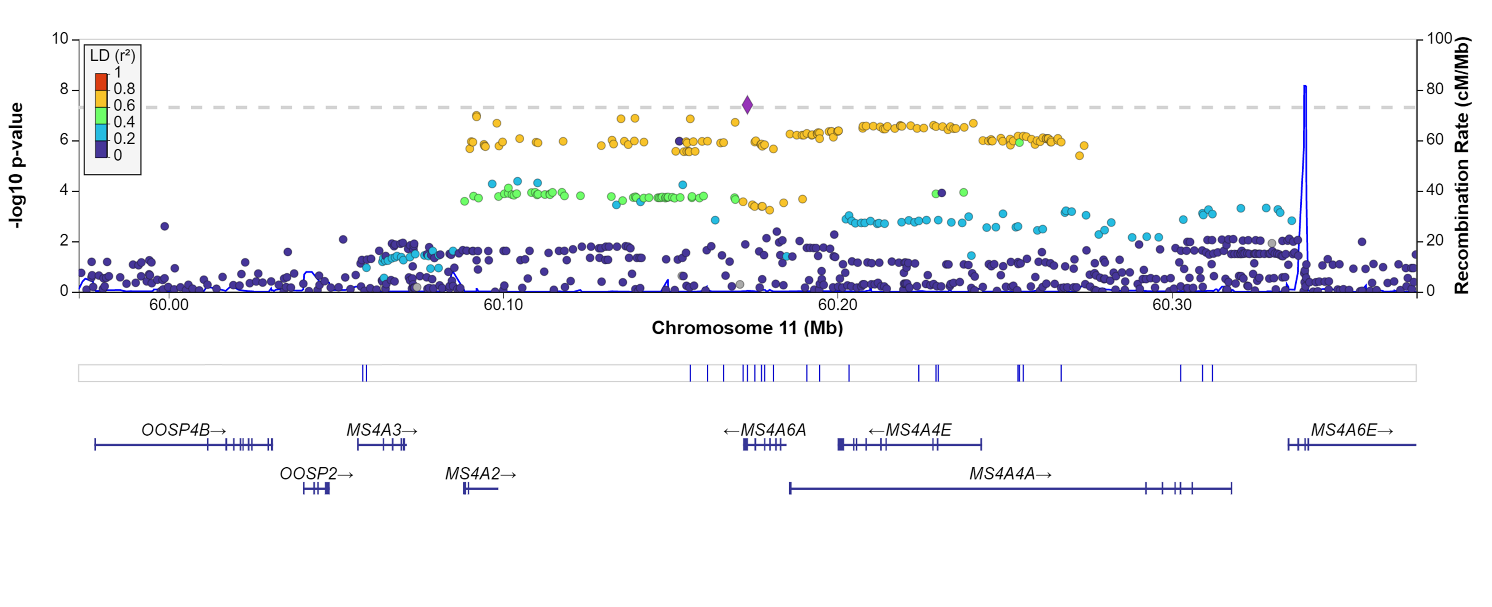
**

**Fig. S8. Manhattan and QQ plots for pleiotropy GWAS in pairs of cognitive domain scores in the total sample**

**(a) Executive function and language**


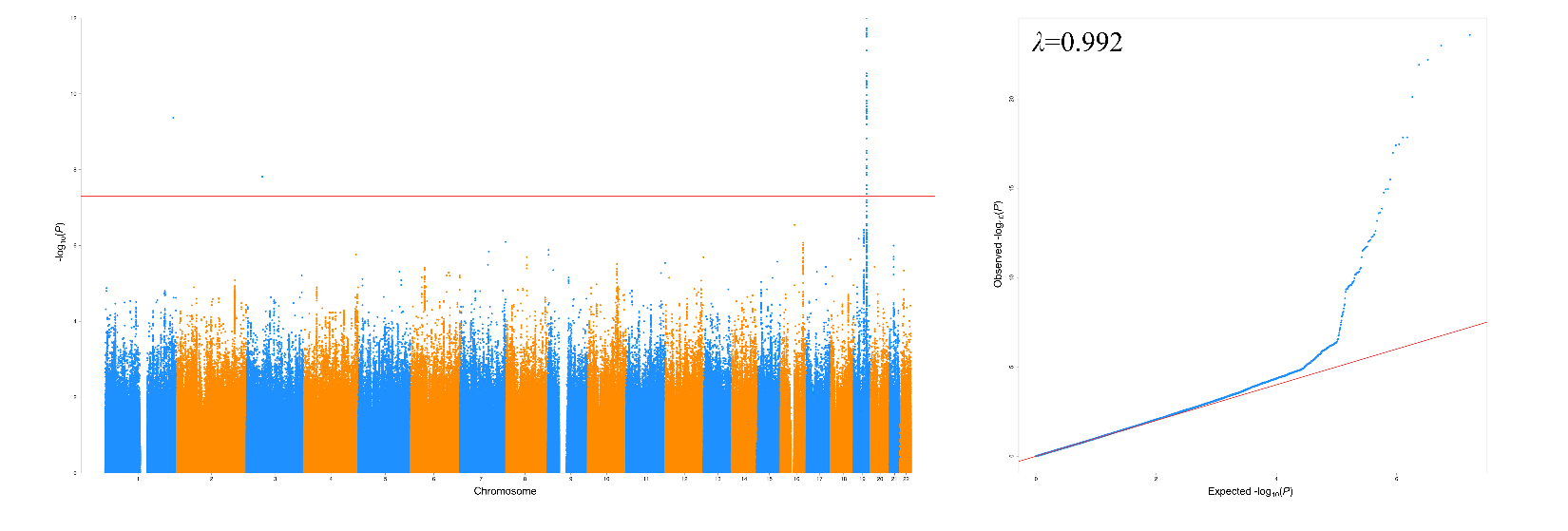


**(b) Executive function and memory**


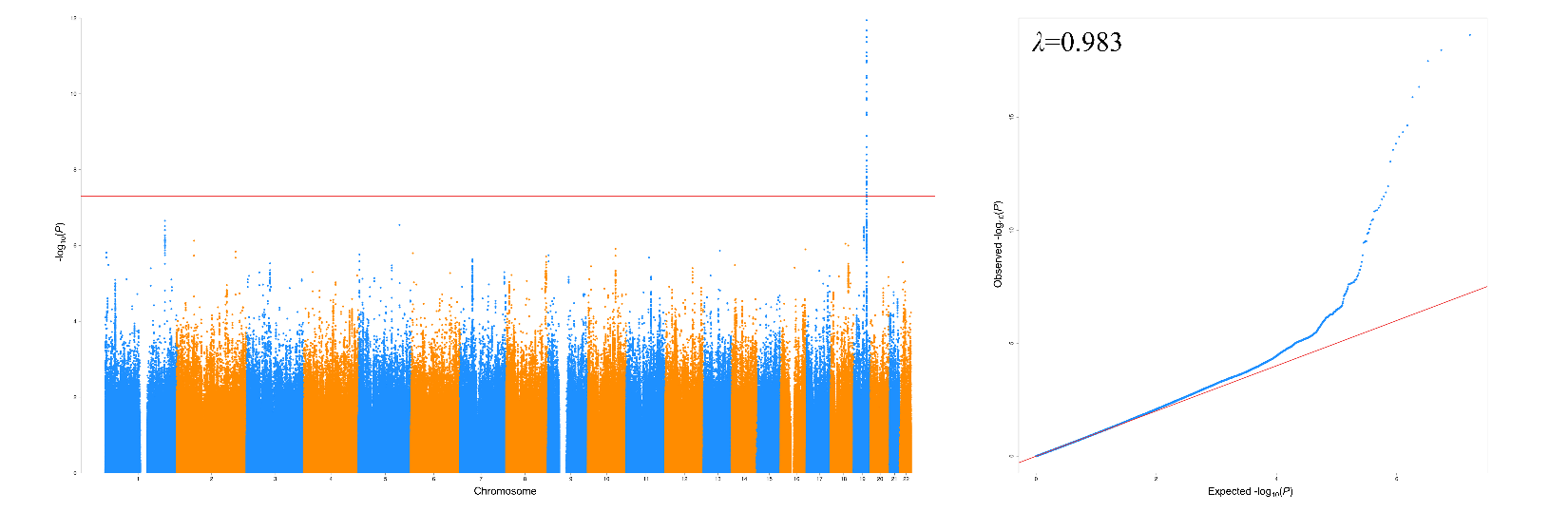


**(c) Language and memory**


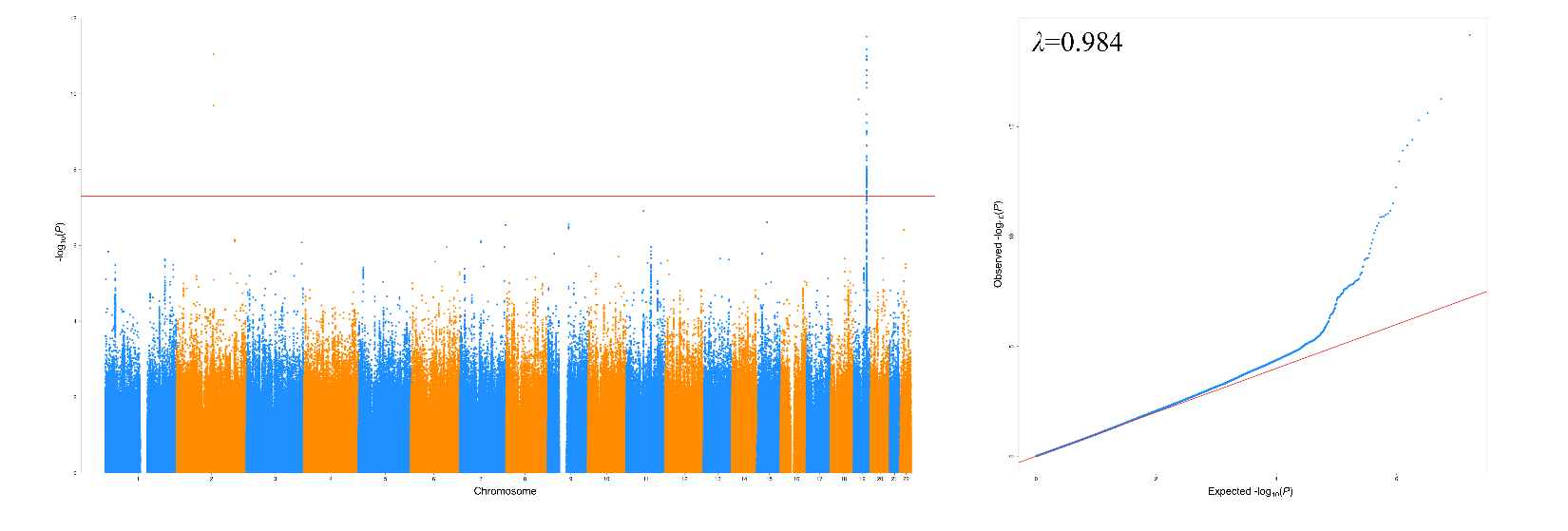


**Fig. S9. Manhattan and QQ plots for pleiotropy GWAS in pairs of cognitive domain scores in the clinic-based cohorts**

**(a) Executive function and language**


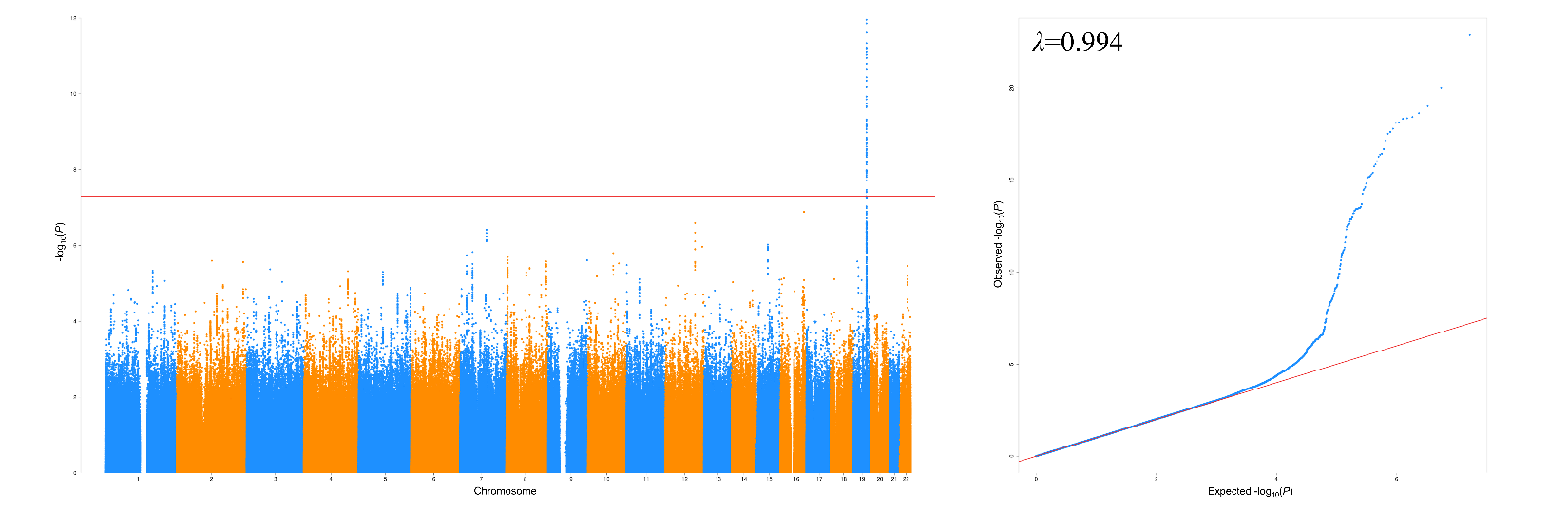


**(b) Executive function and memory**


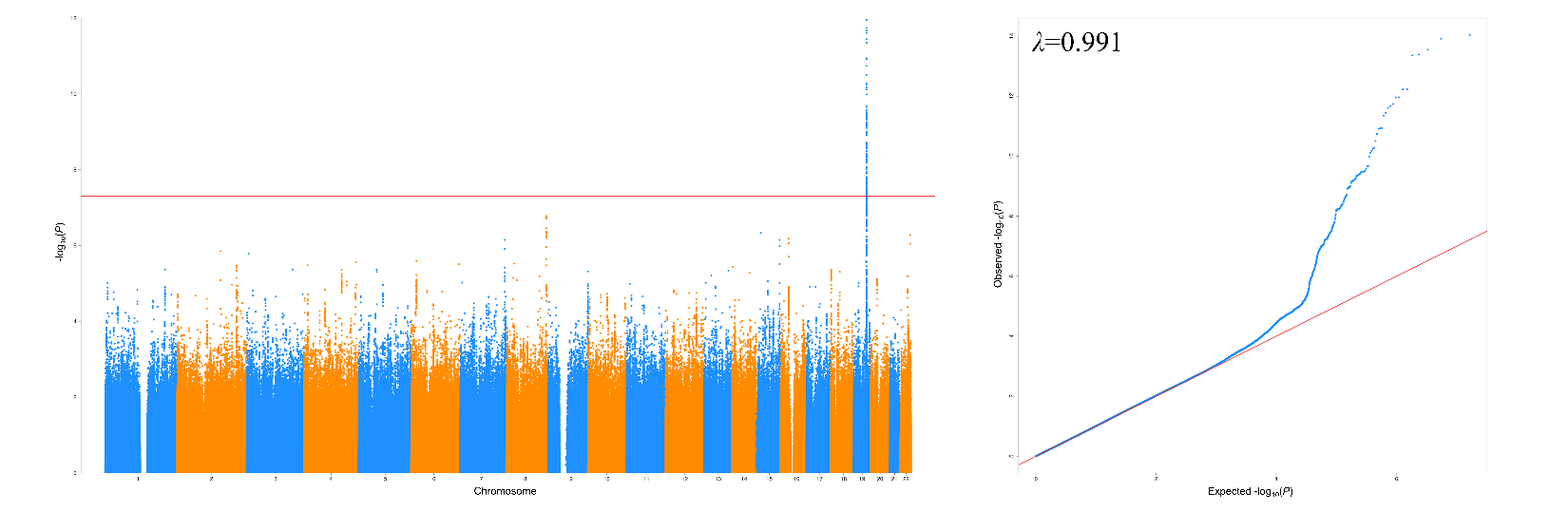


**(c) Language and memory**


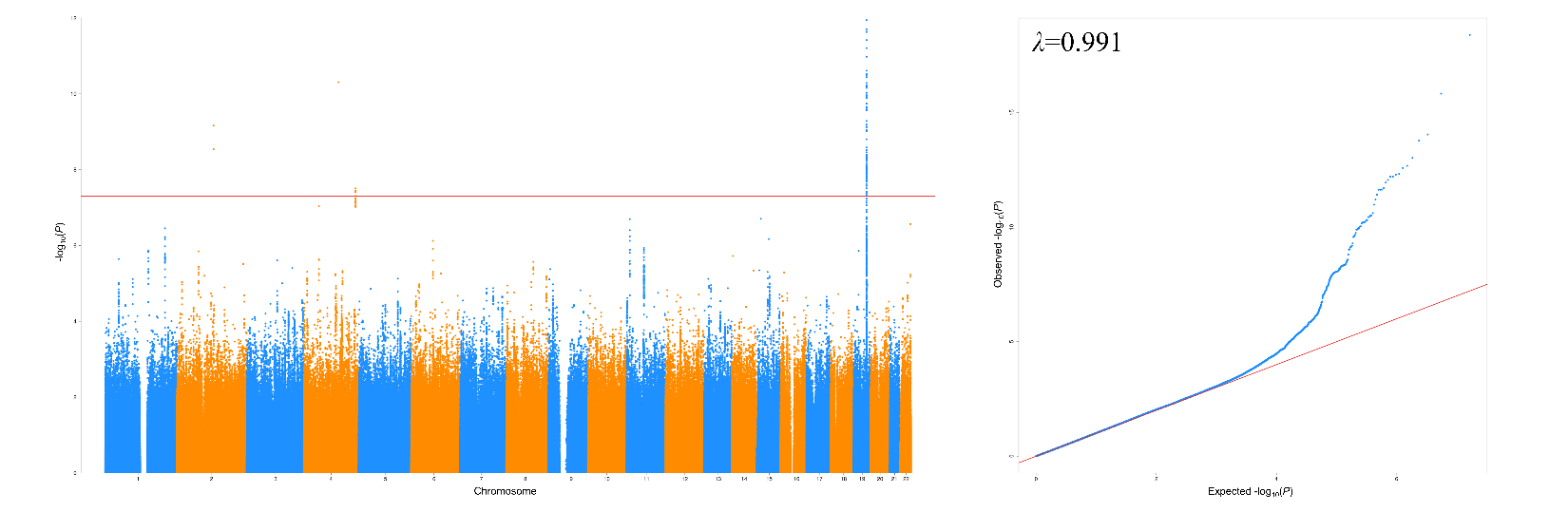


**Fig. S10. Manhattan and QQ plots for pleiotropy GWAS in pairs of cognitive domain scores in the community-based cohorts**

**(a) Executive function and language**


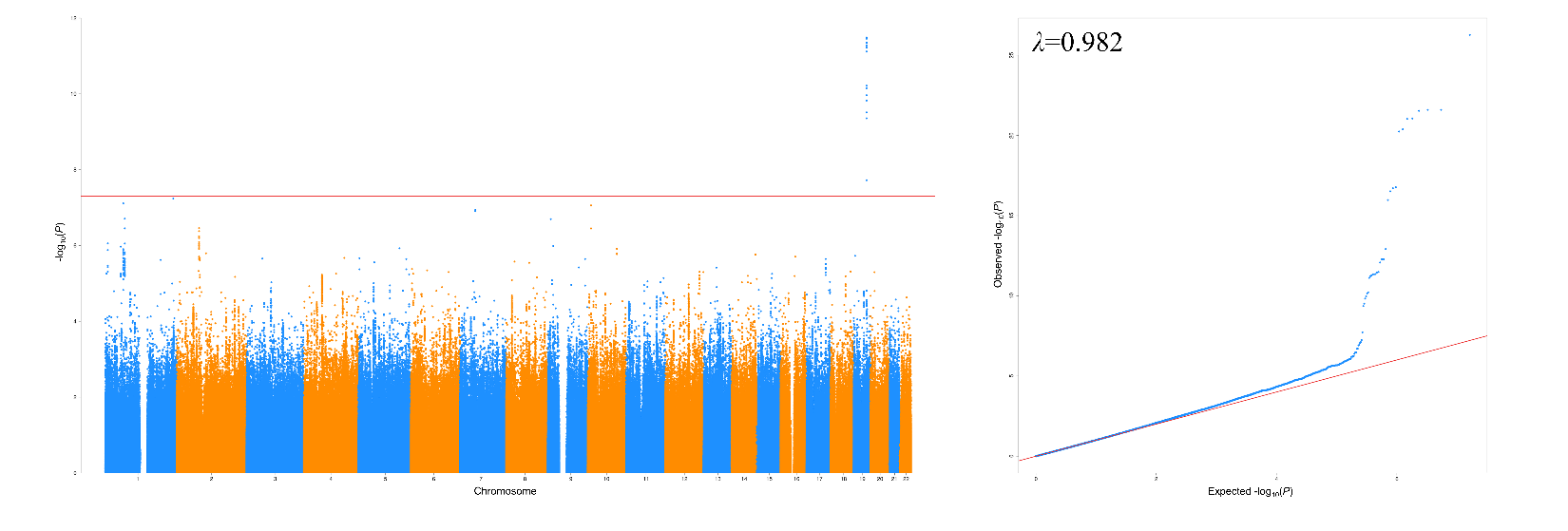


**(b) Executive function and memory**


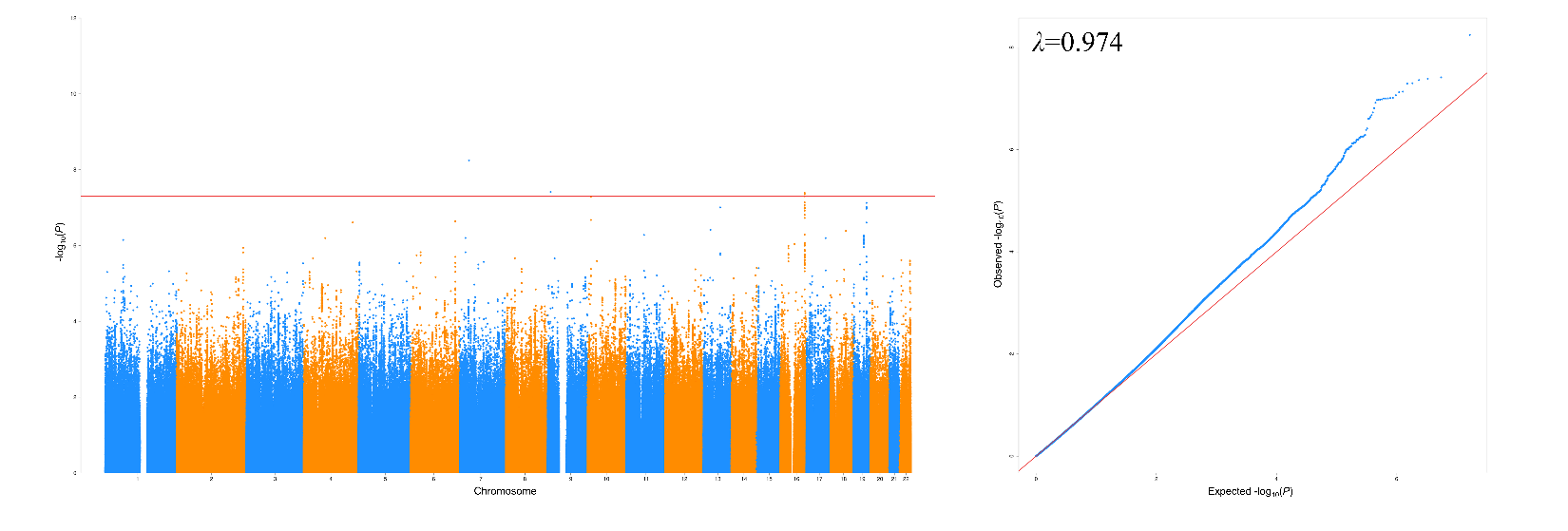


**(c) Language and memory**


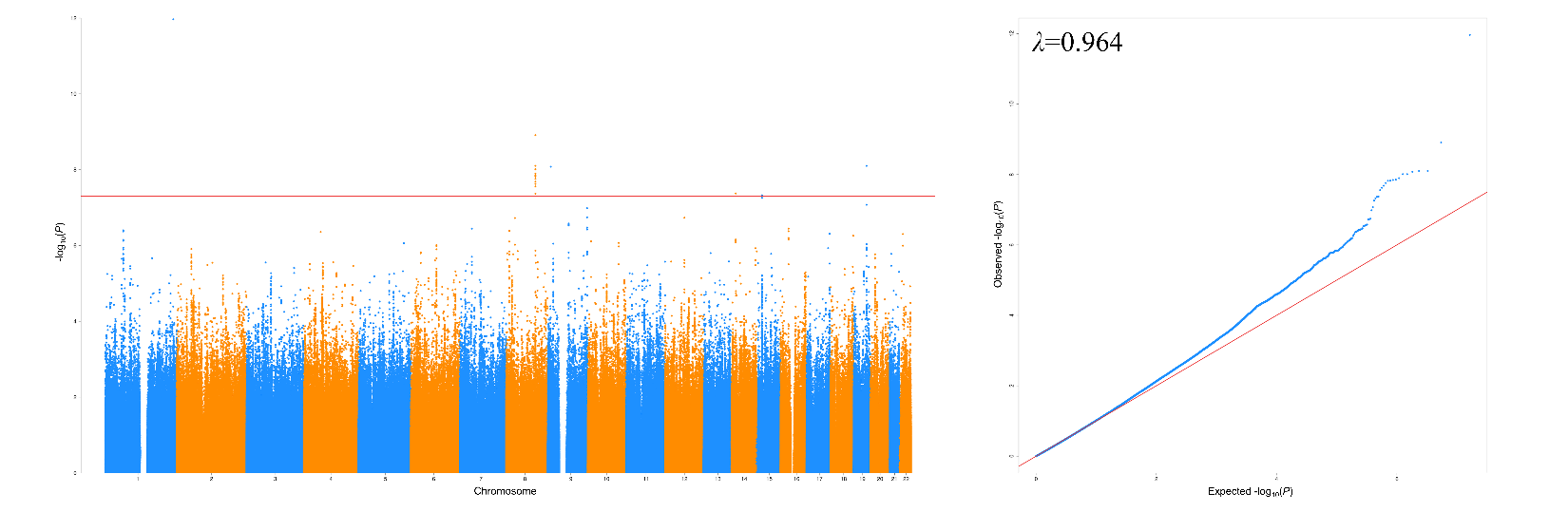


**Fig. S11. Locus Zoom plots showing pleiotropy of SNPs in the *BIN1* region with language and memory.** The SNP with the lowest *p*-value (rs6733839) is indicated with a purple diamond. Computed estimates of linkage disequilibrium (*r*^2^) of SNPs in the region with rs6733839 are color-coded according to the key. Vertical blue lines indicate locations of high recombination rates. Locations of genes in the region are shown below the diagram.

**(a) Total sample**


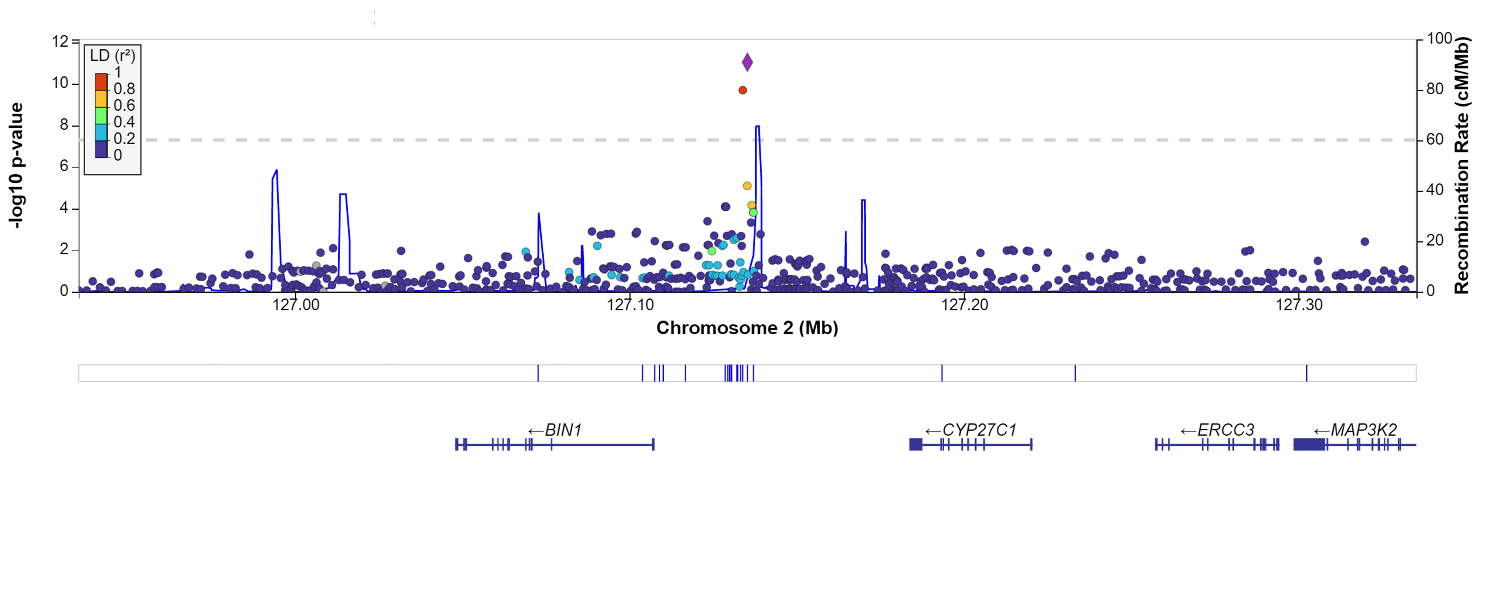


**(b) Clinic-based cohorts**

**
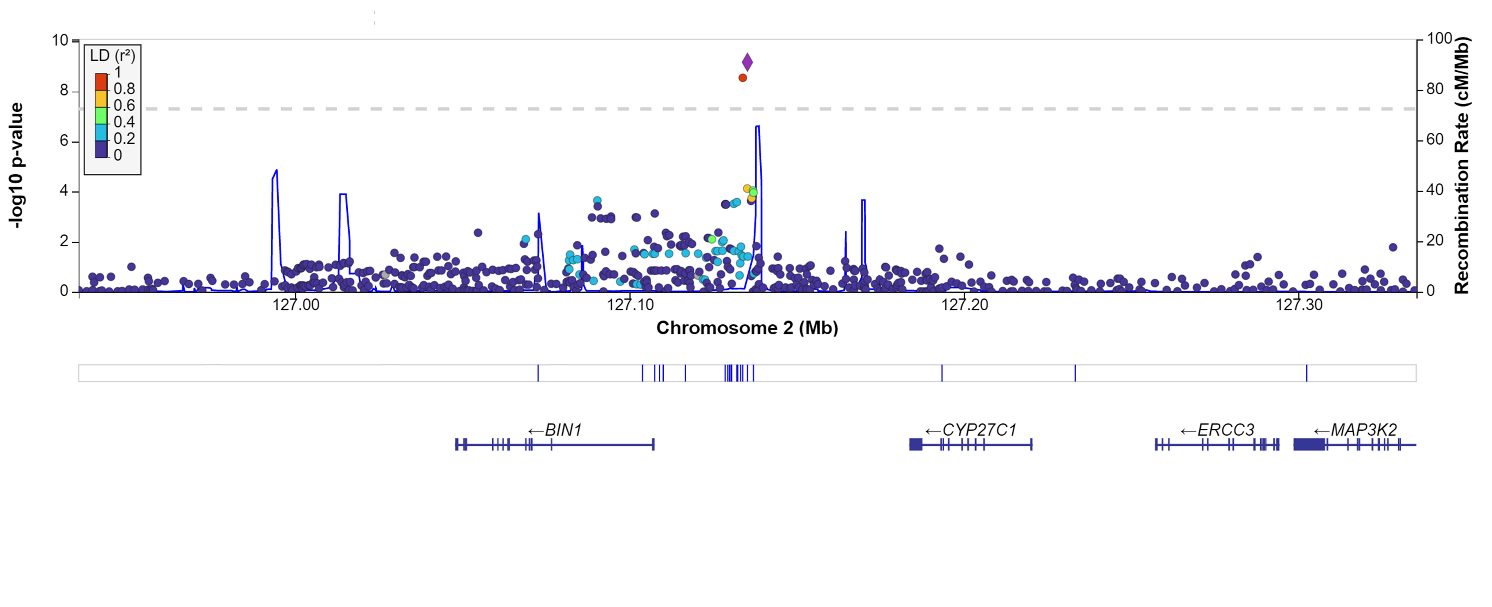
**
